# A Printed Microscopic Universal Gradient Interface for Super Stretchable Strain‐Insensitive Bioelectronics

**DOI:** 10.1002/adma.202414203

**Published:** 2025-02-09

**Authors:** Kaidong Song, Jingyuan Zhou, Chen Wei, Ashok Ponnuchamy, Md Omarsany Bappy, Yuxuan Liao, Qiang Jiang, Yipu Du, Connor J. Evans, Brian C. Wyatt, Thomas O’ Sullivan, Ryan K. Roeder, Babak Anasori, Anthony J. Hoffman, Lihua Jin, Xiangfeng Duan, Yanliang Zhang

**Affiliations:** ^1^ Department of Aerospace and Mechanical Engineering University of Notre Dame Notre Dame IN 46556 USA; ^2^ Chemistry and Biochemistry Department University of California Los Angeles Los Angeles CA 90095 USA; ^3^ Department of Mechanical and Aerospace Engineering University of California Los Angeles Los Angeles CA 90095 USA; ^4^ Department of Electrical Engineering University of Notre Dame Notre Dame IN 46556 USA; ^5^ School of Materials Engineering Purdue University West Lafayette IN 47907 USA

**Keywords:** 3D printing, stretchable bioelectronics, universal gradient interface

## Abstract

Stretchable electronics capable of conforming to nonplanar and dynamic human body surfaces are central for creating implantable and on‐skin devices for high‐fidelity monitoring of diverse physiological signals. While various strategies have been developed to produce stretchable devices, the signals collected from such devices are often highly sensitive to local strain, resulting in inevitable convolution with surface strain‐induced motion artifacts that are difficult to distinguish from intrinsic physiological signals. Here all‐printed super stretchable strain‐insensitive bioelectronics using a unique universal gradient interface (UGI) are reported to bridge the gap between soft biomaterials and stiff electronic materials. Leveraging a versatile aerosol‐based multi‐materials printing technique that allows precise spatial control over the local stiffnesses with submicron resolution, the UGI enables strain‐insensitive electronic devices with negligible resistivity changes under a 180% uniaxial stretch ratio. Various stretchable devices are directly printed on the UGI for on‐skin health monitoring with high signal quality and near‐perfect immunity to motion artifacts, including semiconductor‐based photodetectors for sensing blood oxygen saturation levels and metal‐based temperature sensors. The concept in this work will significantly simplify the fabrication and accelerate the development of a broad range of wearable and implantable bioelectronics for real‐time health monitoring and personalized therapeutics.

## Graphical abstract

Stretchable bioelectronics conforming to dynamic body surfaces enable high‐fidelity physiological monitoring but often suffer from motion artifacts. All‐printed strain‐insensitive bioelectronics are presented using a universal gradient interface (UGI) created via aerosol‐based multi‐materials printing (AMMP). The UGI minimizes local strain changes under 180% stretch, supporting artifact‐free sensors for health monitoring and advancing wearable and implantable bioelectronics for personalized healthcare.

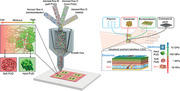

## 1 Introduction

Stretchable bioelectronics hold the key for creating implantable and on‐skin devices that can conform to nonplanar and dynamic human body surfaces,^[^ ^1^, ^2^ ^]^ which are indispensable for high‐fidelity continuous monitoring of physiological signals and personalized healthcare.^[^ ^3^, ^4^, ^5^, ^6^ ^]^ A major challenge in these emerging technologies is the significant mismatch between the properties of soft biological systems and stiff electronic devices. To realize stable integration with biological tissues with seamless conformal interfaces, it is crucial to ensure that the physiological deformation of soft tissues is not impeded by the bioelectronics and that the deformation of the tissues does not interfere or impair the functionality of the electronic devices.

To enhance the stretchability of electronic devices, considerable efforts have been devoted to the development of intrinsically stretchable materials that preserve electrical connectivity under extensive deformation.^[^ ^7^, ^8^, ^9^, ^10^, ^11^, ^12^, ^13^, ^14^ ^]^ However, the electronic properties of these stretchable materials are generally inferior to the conventional non‐stretchable materials, and their properties often drift during repeated stretching cycles.^[^ ^15^, ^16^ ^]^ Alternatively, geometrical design strategies (e.g., serpentines and wrinkles) have been employed to enhance the stretchability of thin metal films by converting strain into structural bending, achieving high conductivity and stability within a specific strain limit.^[^ ^8^, ^17^, ^18^ ^]^ However, these designs demand specialized fabrication techniques for patterning on elastomeric substrates and extra space for the necessary bends and folds, potentially leading to larger devices and lower packing density of components, reducing areal efficiency.^[^ ^19^ ^]^ Additionally, the microscopic wrinkles often prevent the conformal interface that is critical for signal transduction.

Another strategy for stretchable electronics is to generate a nonuniform strain distribution by using substrates comprising strain‐free and strain‐absorbing regions.^[^ ^20^, ^21^ ^]^ Recent developments in strain‐engineered elastomeric substrates using island‐bridge structures have been employed for stretchable electronics.^[^ ^4^, ^22^ ^]^ Such designs include embedding stiff platforms within soft substrates,^[^ ^11^, ^23^, ^24^, ^25^ ^]^ introducing soft interlayer,^[^ ^26^ ^]^ utilizing core/shell packages with ultralow modulus cores,^[^ ^27^ ^]^ and incorporating liquid‐filled cavities in the soft substrate^[^ ^28^ ^]^ to protect functional components from strain. Nonetheless, these systems all involve stiff‐soft interfaces with a sharp change of mechanical properties, which are prone to mechanical failures due to high stress concentrations.^[^ ^24^, ^25^, ^29^ ^]^ To this end, it is essential to design interfaces that can bridge the gap between stiff components and soft surfaces, ensuring they can withstand strains without compromising performances.

Nature provides an elegant solution to bridge the gap between soft and stiff materials via a functionally graded interface of gradient stiffness, allowing stiff components to adhere intimately with soft materials while minimizing the interfacial stress and avoiding delamination. This strategy has been adopted to produce functionally graded interfaces via various fabrication techniques such as adjusting polymer chain cross‐linking degree by tuning the photocuring,^[^ ^30^ ^]^ solvent‐welding of multiple layers with varying reinforcement levels,^[^ ^29^ ^]^ multi‐material extrusion printing,^[^ ^31^ ^]^ and adjusting local phase transitions in hydrogels.^[^ ^32^ ^]^ However, the majority of these mechanically graded materials only have a 1D property gradient and relatively coarse stiffness gradient resolution (i.e., millimeter scale). Major challenges still exist in fabricating these interfaces with smooth gradient stiffness distributions with high spatial resolution and realizing precise control over the local compositions and properties across all three dimensions.

**Figure 1:**
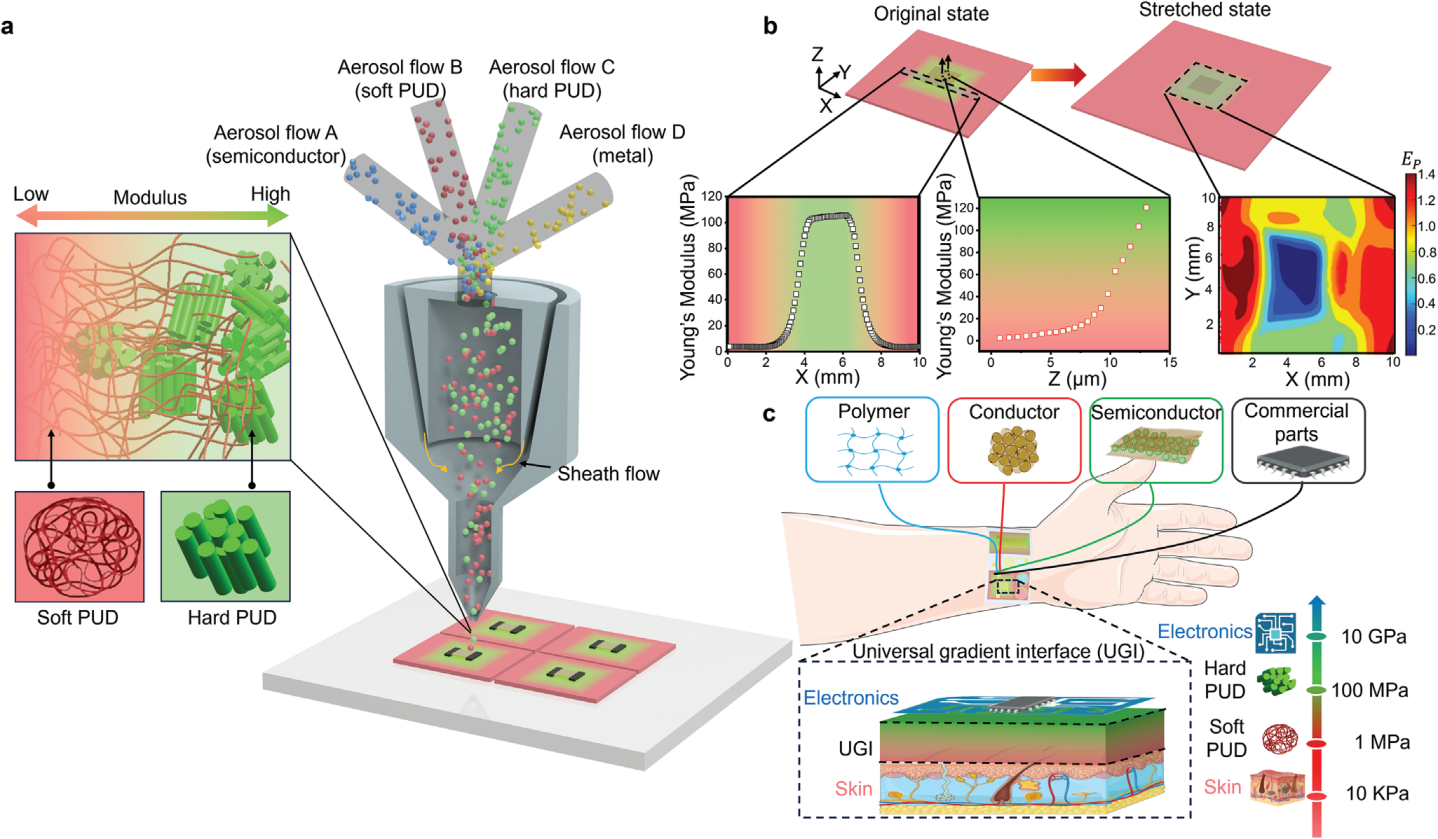
Stretchable devices enabled by universal gradient interfaces (UGIs) printed using aerosol‐based multi‐materials printing (AMMP). a) Scheme for one‐step AMMP to print both UGI and functional devices. b) Schematic illustration of the original and stretched states of a 3D gradient UGI. Young's modulus distributions of the UGI and the maximum principal Lagrange strain map of the 3D UGI under 100% bi‐axial stretch ratio. c) Schematic illustration of the wide range of materials that can be integrated with the UGI.

Herein, we report all‐printed super stretchable strain‐insensitive bioelectronics using a unique universal gradient interface (UGI) to bridge the gap between soft tissues and stiff electronic materials. As shown in **Figure** **1**, the UGI with submicron stiffness gradient is printed using an aerosol‐based multi‐materials printing (AMMP) by in situ modulating the mixing ratio of multiple inks of vastly different properties in a single nozzle, realizing precise control of the local properties along all the three dimensions. Its unique capability for in situ aerosol‐phase mixing and printing enables instantaneous tuning of material compositions, a feature difficult to achieve with conventional multi‐material printing methods that rely on liquid–liquid or solid–solid feedstocks. A variety of semiconducting and metallic 2D/1D/0D nanomaterials can be printed on the UGI to form electronics with remarkable strain insensitivity and negligible resistivity changes under a high stretch ratio of up to 180%. The all‐printed devices deliver high signal quality and near‐perfect immunity to motion artifacts for on‐skin health monitoring of blood oxygen saturation levels, temperature, and pulses. Our facile approach greatly simplifies the fabrication of super‐stretchable bioelectronics, enabling harmonious integration of a broad range of functional devices with biological systems for emerging applications such as wearable/implantable devices, soft/hybrid robotics, and human/machine interfaces, etc.

## 2 Universal Gradient Interfaces (UGIs) Using AMMP

AMMP allows to directly print the UGI with gradient modulus with submicron spatial resolution. The AMMP approach starts by atomizing two (or multiple) inks into aerosols that consist of microscale droplets. These aerosols are then mixed in situ in a single nozzle and focused using a co‐flowing sheath gas prior to deposition (Figure 1a). The AMMP enables precise control over local material composition by dynamically modulating the mixing ratio of different aerosolized inks. Taking advantage of our multi‐material printing process, the UGI and functional devices can be printed all in a single nozzle, significantly improving fabrication efficiency.

In this work, the UGI is printed by mixing two polyurethane dispersions (PUDs) with drastically different moduli. The soft PUD contains flexible polyester polyol segments with low cross‐linking, resulting in a lower modulus and higher elongation at break. Conversely, the stiff PUD is composed of hard segments derived from methylene diphenyl diisocyanate (MDI), leading to a higher modulus and lower elongation at break. This combination allows the elastic modulus of the gradient material system to span two orders of magnitude, ranging from 1 MPa (soft PUD) to over 100 MPa (stiff PUD) (Figure 1b). Upon stretching the UGI, the deformation concentrates in low modulus regions (red color) (Figure 1b), which makes the high modulus region (green color) a strain‐isolation region, on which printing the functional electronics is beneficial. Our AMMP approach achieves a high resolution of ≈500 nm along the deposition thickness (Z direction) and 20 µm in the X‐Y plane (Figures S1 and S2, Supporting Information). For demonstration, a 3D gradient UGI is displayed where digital image correlation (DIC) reveals a 2.5% maximum principal Lagrange strain in stiff areas, while the whole 3D gradient UGI is under 100% equi‐biaxial stretch, showcasing its superior strain isolation capability (Figure 1b). This method effectively bridges the gap between soft tissues and a wide variety of stiff materials commonly used in electronic/sensing devices (Figure 1c).

**Figure 2:**
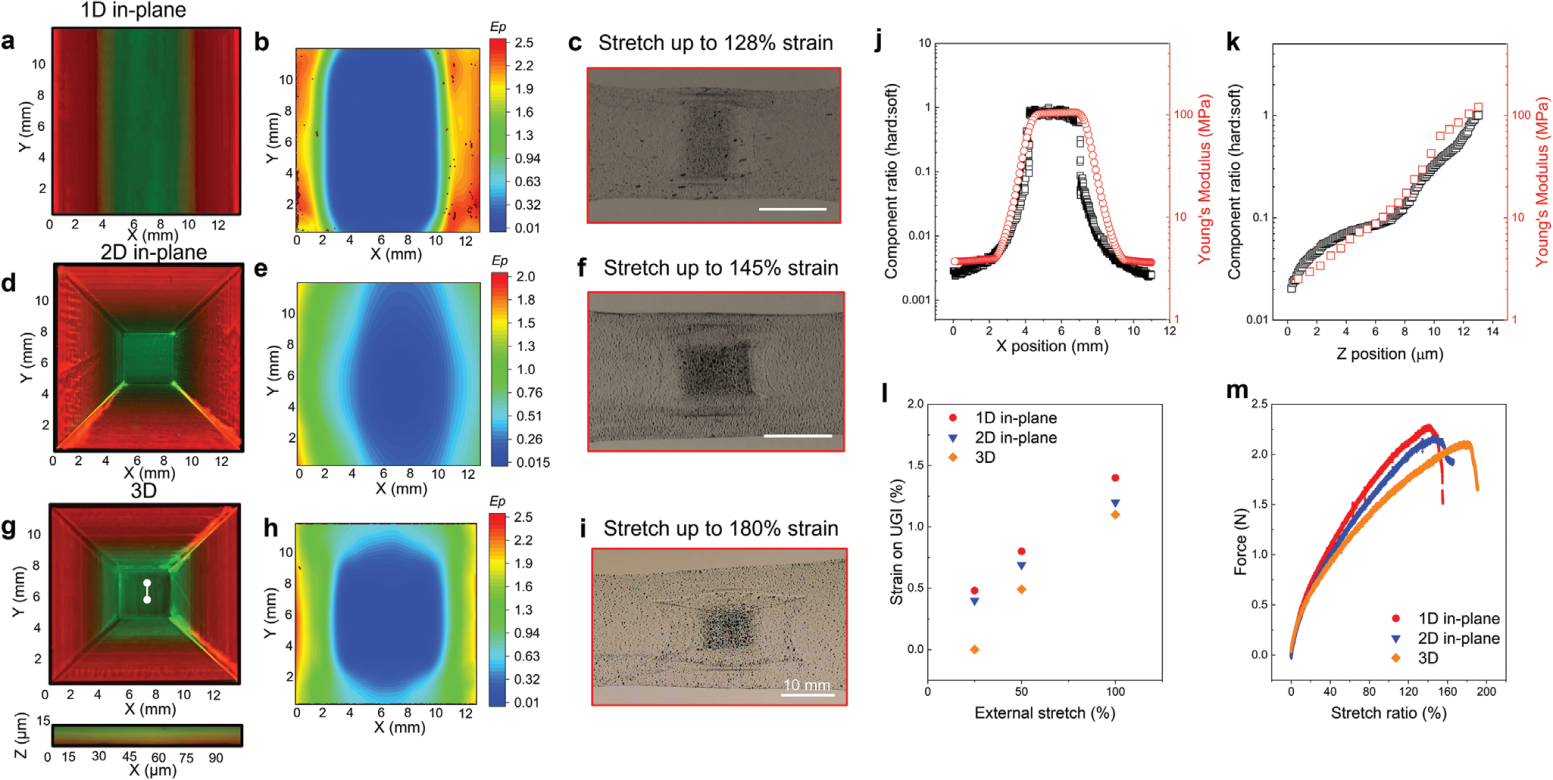
Investigation of different UGI designs. Gradient design, maximum principal Lagrange strain map (*E_p_*) under 100% uniaxial stretch ratio, and optical image under maximum stretch ratio for a–c), 1D in‐plane gradient, d–f), 2D in‐plane gradient, and g–i), 3D gradient. j), Composition and modulus distributions vs. X‐positions for 1D in‐plane gradient; k), Composition and modulus distributions vs. Z‐positions for 3D gradient. l), Maximum principal Lagrange strain values on the center of the stiff region vs. external stretch for different UGI designs. m), tensile test results for different UGI designs.

To validate the strain‐isolation performance of our method, we first printed UGIs to assess the effect of stiffness gradients on strain and stress distributions under varying loading conditions. Five different UGI designs were printed, including non‐gradient, 1D in‐plane, 2D in‐plane, 1D out‐of‐plane, and 3D gradients (**Figure** **2**a,d,g; Figures S3 and S4, Supporting Information). Gradient mixing was visualized using fluorescent imaging by incorporating red and green fluorescent dyes into the two PUD inks. The white line in Figure 2g indicates the position of the lower graph showing the out‐of‐plane (or z‐direction) distribution of the two polyurethanes. In Figure 2d,g, the brighter coloration along the diagonal position is attributed to the designed concentric infill printing paths, which result in slightly increased ink deposition in that area. However, this minor variation in ink deposition has negligible impact on UGI performance. Through in situ modulation of the material composition, these designs lead to different site‐specific elastic moduli distributions (Figure 2j,k; Figure S4, Supporting Information). Since the X‐direction gradient design is identical for both the 1D and 2D in‐plane gradient samples, their composition and modulus distributions along the X direction are the same. The top surface maximum principal Lagrange strain maps obtained from DIC for samples under 100% uniaxial global stretch show significant variations in strain distribution among different designs, particularly near the stiff regions and stiff/soft interfaces (Figure 2b,e,h; Figures S4 and S5, Supporting Information). Here, a 100% uniaxial stretch ratio indicates the material is stretched to two times of its original length ($\frac{L-L_{0}}{L_{0}}=100\%$, L is the length after stretch and *L*_0_ is the length before stretch). Finite Element Analysis (FEA) models were also created, presenting strain distributions consistent with experiments. These models provide valuable insights into stress/strain distribution under varying loading conditions (Figures S6,S9, and S10, Supporting Information). The non‐gradient design exhibits stress and strain concentration at the soft‐stiff interfaces (Figures S4–S6,S9, and S10, Supporting Information). Due to the sharp modulus transition, significant strain mismatch occurs at the interface during loading, consequently resulting in elevated stress level. In this way, a failure is likely to initiate early, occurring at ≈48% uniaxial strain (Figures S4 and S5, Supporting Information). Conversely, the UGI designs effectively reduce local stress and strain concentrations arising from the sharp contrast of elastic moduli between the stiff and soft components. Under 100% uniaxial tension, maximum principal Lagrange strains remain as low as 1.4%, 1.2%, and 1.1% for 1D in‐plane, 2D in‐plane, and 3D gradients in the stiff areas, respectively (Figure 2l). The strain progressively increases along the direction of decreasing modulus (Figure 2b,e,h; Figure S5, Supporting Information), as softer materials undergo greater deformation under the identical external loads. Notably, the smooth strain transitions in 2D in‐plane, and 3D UGIs significantly mitigate stress concentration (Figures S6,S9, and S10, Supporting Information). Figure S5 (Supporting Information) shows excellent strain isolation for the 3D UGI, where the strain in the stiff region is as small as 2.7% while the uniaxial stretch ratio approaches 180%.

Furthermore, the initial failure points differ across designs. Non‐gradient design tends to fail prematurely at the stiff‐soft interface due to high stress and strain concentrations caused by the sharp modulus changes (Figures S4 and S5, Supporting Information). In contrast, UGIs exhibit failure within the soft region when the strain exceeds the intrinsic limit of the soft polyurethane (Figure 2c,f,i; Figure S6, Supporting Information). These gradient designs effectively distribute stress and strain across the stiff‐soft transition region, thereby significantly enhancing global failure resistance. Figure 2m and Figure S8 (Supporting Information) display force‐displacement curves for different designs. The above results underscore the crucial role of stress distribution in crack initiation and illustrate the significance of gradient design that facilitates more uniform stress distribution.

## 3 Versatility of UGI to Enable Strain‐Insensitive Stretchable Materials and Devices

**Figure 3:**
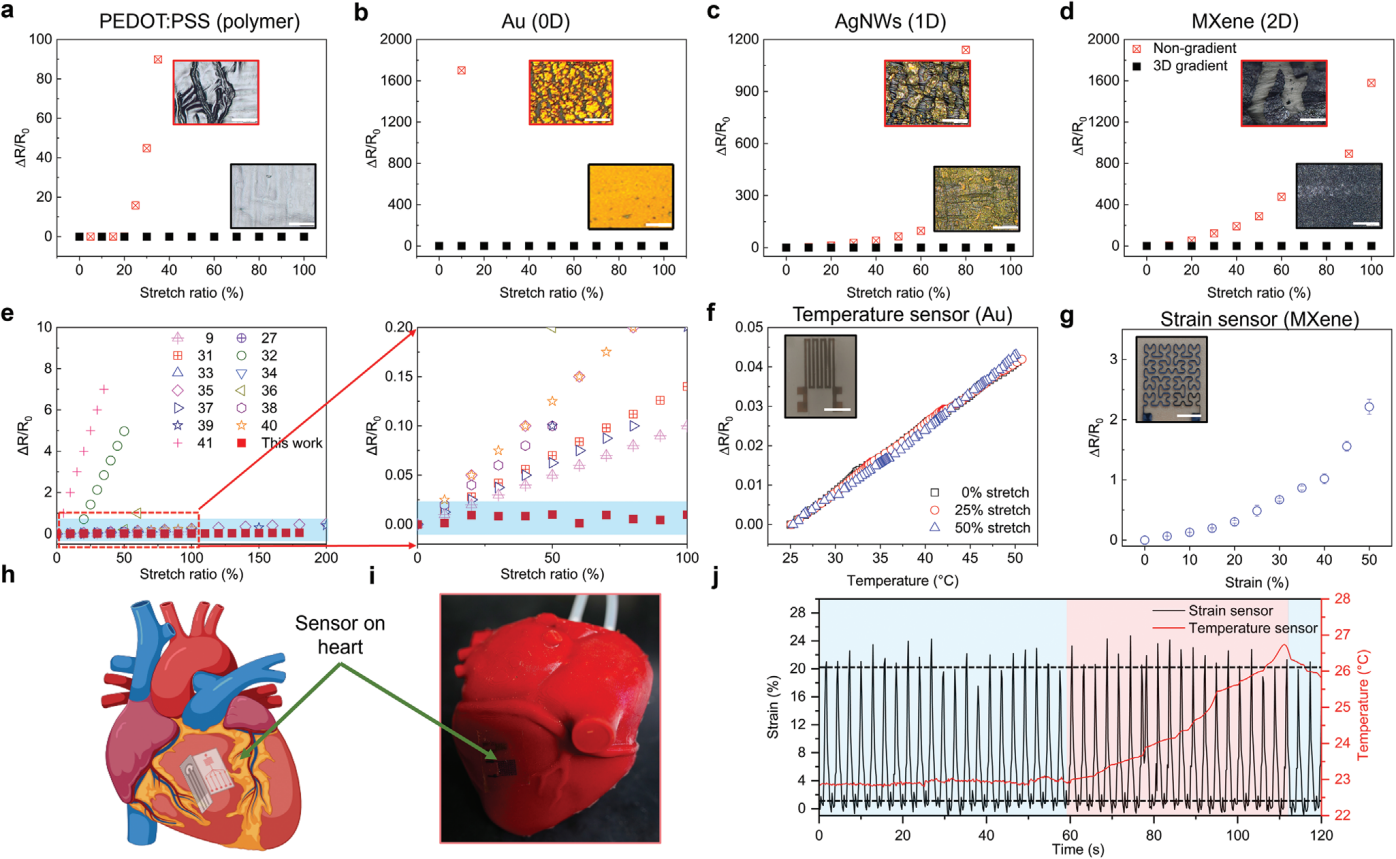
Demonstration of the strain‐insensitive effect of various conductive materials printed on the UGI. Relative resistance change (RRC) vs. stretch ratio for a) PEDOT: PSS, b) Au, c) AgNWs, and d) MXene. Scale bar, 100 µm. e) Comparison of RRC in this work with previously reported stretchable electronics.^[^ ^11^, ^29^, ^33^, ^34^, ^35^, ^36^, ^37^, ^38^, ^39^, ^40^, ^41^, ^42^, ^43^ ^]^ f) Temperature sensor printed on the UGI, and g) strain sensor printed on soft polyurethane. Scale bar, 4 mm. h) Schematic and i) optical image of multimodal temperature and strain sensor on an artificial heart model. j) Real‐time strain and temperature sensing during the periodic diastole and systole when the heart model is under stable temperature followed by a transient heating.

To demonstrate the versatility and universal applications of the UGI, we printed conductive polymer films of PEDOT: PSS, 0D gold nanoparticle, 1D silver nanowires (AgNWs), and 2D MXene on the 3D UGI and subjected them up to 100% uniaxial stretch ratio (see Table S3 and S4, Supporting Information for the detailed fabrication strategies). The printed Au film was sintered using a photonic flash sintering process detailed in Figure S11 (Supporting Information). The conductive films printed on homogeneous soft PU substrates were also tested for comparison. As shown in **Figure** **3**a–d, all the conductive films on UGI show no visible cracks at a 100% stretch ratio, with the relative resistance change (RRC) (ΔR/R_0_) < 4.2%, whereas those on homogeneous substrates showing significant RRC on increases and microcracks leading to electrical failure. To validate the UGI's mechanical repeatability and stability, we performed a 1,000‐cycle tensile test at a 50% uniaxial stretch ratio on a 3D gradient UGI. The results, detailed in Figure S13 (Supporting Information), show a low RRC of less than 1.6%, demonstrating the 3D UGI's durability and resilience under cyclic loading. These results highlight the superior strain isolation properties of the UGI design compared to state‐of‐the‐art stretchable electronics (Figure 3e).^[^ ^11^, ^29^, ^33^, ^34^, ^35^, ^36^, ^37^, ^38^, ^39^, ^40^, ^41^, ^42^, ^43^ ^]^

Next, we explored the printing of electronic devices and sensors on the UGI. A stretchable Au‐based resistive temperature sensor is printed on the 3D UGI (Figure S14, Supporting Information), demonstrating a sensitivity of 1.6 × 10^−3^ °C^−1^ across a 25 to 50 °C range, with stable sensitivity during stretching (Figure 3f). Tools for cardiac physiological mapping are indispensable for the clinical identification and mechanistic understanding of excitation–contraction coupling, metabolic dysfunction, arrhythmia, and other conditions. Employing an artificial heart model, we evaluated the real‐time performance of our sensors under dynamic conditions. To monitor the heart beating during diastole and systole, an MXene‐based strain sensor was printed on the homogeneous soft substrate (Figure 3g). The strain and temperature sensors were then affixed to an artificial heart (Figure 3h,i), which pulsates every 2 s. Our measurements show that the strain sensor can catch the periodic diastole and systole process (Figure 3j). Concurrently, the temperature sensor records temperature changes over time, maintaining high stability under repeated strain cycles (<1.15% resistance change during consistent temperature conditions). These results indicate that the UGI provides a robust platform to monitor real‐time signals in a dynamic environment.

**Figure 4:**
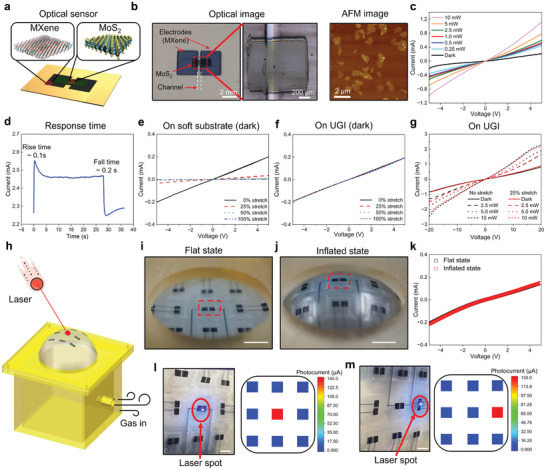
Stretchable photodetectors. a) Schematic, b) optical images and Atomic force microscopy (AFM) image^[^ ^44^ ^]^ showing a printed photodetector on the UGI. c) Photocurrent vs. bias voltage under different illumination powers. d) Time‐dependent photocurrent at an applied DC bias of 5V. Dark current vs. bias voltage under different stretch ratios on e) soft polyurethane, and f) UGI. g) Photocurrent vs. bias voltage under different illumination powers when the UGI is under no stretch and 25% stretch h) Schematic of a deformable 3D photodetector array. i,j) Optical images of the 3D photodetector under a flat and inflated state. k) Dark current vs. bias voltage during flat and inflated state. l‐m) The photocurrent mapping under upright illumination and tilted‐angle illumination. The radius of the hemisphere is 30 mm. Scale bar, 5 mm.

In addition to the stretchable resistance‐based sensor, the UGI is employed to develop more sophisticated stretchable optoelectronic devices utilizing printed semiconducting 2D MoS_2_, which transitions from an indirect to a direct bandgap in a monolayer regime and thus is ideal for optoelectronic applications in the atomically thin limit.^[^ ^45^ ^]^ As illustrated in **Figure** **4**a,b, the MoS_2_ inks were printed as the channel material between the two printed MXene electrodes (Figure S17, Supporting Information), exhibiting a power‐law relationship (*R* ∝ *P^γ^*, *γ* = −0.06), resulting in higher responsivities (*R*) at lower powers (*P*) (Figure 4c; Figure S18, Supporting Information). At low laser intensity (0.15 W cm^−2^), the responsivity for the devices reached 0.62 A W^−1^, which is among the highest of previously reported all‐printed photodetectors in the visible light range. Further details on the responsivity calculation are provided in Figure S18 (Supporting Information). Photodetector response times, with rise and fall constants of ≈0.1 and 0.2 s, respectively, reflect the rapid generation and slower recombination of charge carriers facilitated by deep trap states (Figure 4d).^[^ ^46^ ^]^ A trade‐off between response time and MoS_2_ layer thickness is observed (Figure S19, Supporting Information). Our all‐printed photodetectors exhibit relatively fast response times compared to some literature using CVD‐grown and mechanically exfoliated MoS_2_ photodetectors (Figure S19, Supporting Information).^[^ ^47^, ^48^, ^49^ ^]^ The slower response times of CVD‐grown and mechanically MoS_2_ photodetectors, linked to interface traps at the MoS_2_‐SiO_2_ interface, contrast with faster times on polyurethane substrate due to reduced trapping.^[^ ^47^, ^50^ ^]^

To assess the performance of photodetectors on both 3D gradient and soft interfaces, we recorded the dark current under various stretch ratios (Figure 4e,f). Even under 100% uniaxial stretch, the dark current shows <2.9% change on the 3D gradient interface, whereas over 16700% decrease of dark current occurs on the soft substrate. Performance on 3D gradient interfaces under various laser powers and strains demonstrated <2.6% signal drift at 25% stretch (Figure 4g), highlighting the robust strain isolation of the UGI design, crucial for applications in dynamically changing environments like tunable electronic eye cameras.

An array of nine photodetectors was printed and mounted on an inflatable hemispherical structure subjected to variable 2D strains to demonstrate advanced 3D sensing systems. (Figure 4h–j; Figure S20, Supporting Information). The photodetector array exhibits remarkable strain isolation capabilities and <1.9% change of dark current under maximum in‐plane strains of 57% in each direction (Figure 4k). Finite element simulations (Figure S21, Supporting Information) revealed a smooth distribution of maximum principal strain and stress in response to the gradient modulus, mitigating the stress and strain mismatch at the interface between the photodetector and the soft substrate. Notably, the strain in the photodetectors on the UGI remains minimal, indicating the robustness of this design.

The 3D stretchable photodetector array can simultaneously detect both the light intensity and the position of incident light. When laser light (405 nm wavelength) directly strikes the center of the photodetector array, the central detector exhibits a dominant photocurrent of 139.6 µA, with the surrounding eight detectors showing negligible photocurrents (Figure 4l). However, for oblique incident light to the right sensor, the most intense response emerges in the laser‐light‐focused photosensor (right sensor) (Figure 4m; Figure S22, Supporting Information). These findings pave the way for the integration of stretchable photodetectors in versatile 3D sensing applications. Envisioning future advancements, combining these sensors with 3D‐printed laser, visible, and infrared detectors could revolutionize bionic eye development, offering comprehensive all‐angle and all‐wavelength visual capabilities.

## 4 All Printed Strain‐Insensitive On‐Skin Sensors for Continuous Health Monitoring

**Figure 5:**
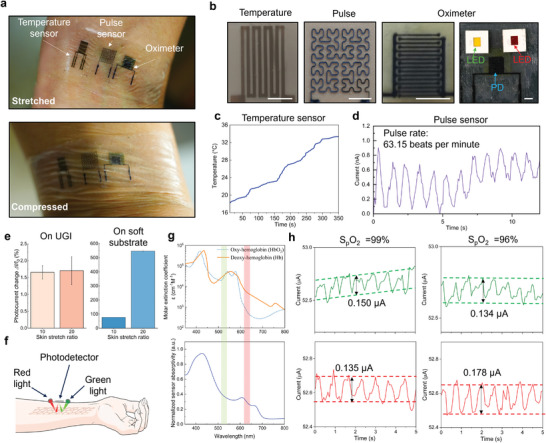
All printed wearable multimodal sensors for health monitoring. a) Optical image of the multimodal sensor. b) Optical images of the temperature sensor, pulse sensor, and oximeter consisting of a printed photodetector (PD) and a green LED and a red LED. Scale bar, 2 mm. Characteristics of the c) temperature sensor and d) pulse sensor. e) Motion artifacts for oximeter printed on UGI and soft substrate. f) Schematic of the oximeter in reflectance mode. g) Molar extinction coefficient of oxygenated (orange solid line) and deoxygenated (blue dashed line) hemoglobin in arterial blood, and absorptivity of oximeter vs. Wavelength. h) Output signal from oximeter with 99% and 96% oxygenation of blood. The green and red lines represent the signals when the green and red LEDs are operated.

The UGI serves as a versatile platform for printing multimodal sensors for on‐skin health monitoring. Here, we demonstrate the harmonious integration of temperature and blood oxygen saturation sensors on the stiff region of the UGI and a pulse sensor on the soft region to detect pulse‐induced strain changes, creating a stretchable system integrated seamlessly with the skin (**Figure** **5**a,b). To ensure robust and reliable measurements while maintaining user comfort, the connection setup for the skin‐mounted sensors is illustrated in Figure S23 (Supporting Information), providing a clear depiction of how the measurements were conducted.

The temperature sensor accurately tracks skin temperature fluctuations, capturing changes from 18.2 to 33.4°C as the participant moves from a cold to a warm environment (Figure 5c). The pulse sensor using printed MXene patterns dynamically records the electrical current variations that correspond with the heartbeat, enabling precise heart rate monitoring by counting peaks over a minute (Figure 5d). Pulse oximetry, a widely utilized non‐invasive method, evaluates tissue oxygen saturation by optically determining the relative concentrations of oxyhemoglobin (HbO_2_) and deoxyhemoglobin (Hb) in blood using photoplethysmography (PPG). Our MoS_2_‐based photodetector is integrated with green and red LED lights (Figure 5f) to create a stretchable pulse oximeter that conforms to the skin. In this setup, reflected light is modulated by the dynamic volumes of arterial blood, affecting light absorption detected by the photodetector. This modulation, correlating with the heart's systolic and diastolic phases, produces a pulsatile optical signal that can be used to determine the blood oxygenation levels (Figure S24, Supporting Information). HbO_2_ and Hb exhibit different absorption rates of oxygenated and deoxygenated hemoglobin at red and green wavelengths (Figure 5g). Blood oxygen saturation (SpO_2_) can be calculated by determining the light absorption ratio at these two wavelengths (Figure S24, Supporting Information). Figure 5h shows the corresponding signals when SpO_2_ decreases from 99% to 96% during a breath hold test.

Motion artifacts pose significant challenges in wearable electronics for health monitoring, leading to potential misinterpretations and severe health implications. To demonstrate the superior immunity to strain/motion artifacts of printed UGI, photodetectors were printed on both UGI and soft substrates, respectively, and then tested on skin with induced strain deformations while recording the signals (Figure S25, Supporting Information). The oximeter on the UGI showed neglectable signal deviation (<1.7% change of measured photocurrent under ≈20% uniaxial strain), as depicted in Figure 5e, demonstrating by far the best performances in reducing motion artifacts among previously reported wearable devices (Table S3, Supporting Information). In contrast, the oximeter on the soft substrate experienced over >547% signal variance (Figure 5e), underscoring the UGI's superior capability for stable biological signal capture amidst skin deformations.

## 5 Conclusion

In summary, the printed universal gradient interfaces (UGIs) with submicron resolution and 3D modulus distributions provide unprecedented strain isolation and a versatile platform for realizing strain‐insensitive stretchable devices with over 180% uniaxial stretchability. The versatility of the aerosol‐based multi‐materials printing (AMMP) method allows for integrating a diverse range of structural and functional materials into stretchable systems with precise control over global device structures and local properties with unparalleled spatial resolution. This UGI platform enables multifunctional stretchable devices for on‐skin sensing of temperature, light, and blood oxygen, which allows truly motion artifact‐free health monitoring. The AMMP has the potential for high throughput printing by deploying a large array of printing nozzles to scale up the printing processes. This innovation paves the way for advanced wearable and implantable devices for real‐time health monitoring and therapeutics, as well as for soft and hybrid robotics with integrated soft‐stiff gradient interfaces that are essential in natural musculoskeletal structures.

## 6 Experimental Section

### General Ink Formulation

Commercially available gold ink (10 wt.% in Xylene) was purchased from UT Dots. AgNWs (1 wt.% in isopropanol) ink was purchased from Nanostructured & Amorphous Materials. PEDOT: PSS (0.8 wt.% in water) were purchased from Sigma–Aldrich. Soft (Baymedix CD 102) and hard PUD (U 9380) inks were obtained from Covestro and Alberdingk Boley, respectively. Depending on the ink type, a small amount of isopropyl alcohol (IPA) was often added as a defoamer to suppress foam formation during ultrasonic atomizing. To avoid aggregation and ensure uniform dispersion, inks were sonicated for 15 min (Fisher Scientific sonicate bath, 9.5L). Typical ink composition can be found in Table S4 (Supporting Information). The concentration of nanomaterial was determined by the drying weight method.

For the preparation of the MoS_2_ ink, a two‐electrode electrochemical cell was used for electrochemical intercalation. A thin piece of cleaved MoS_2_ crystal was clamped by a copper alligator clip as the cathode, and a graphite rod was placed as the anode respectively. Tetraheptylammonium bromide (THAB)/acetonitrile (5 mg mL^−1^) served as the electrolyte. The electrochemical intercalation was conducted under a voltage of 7.5 V for 1 h. Afterward, the as‐intercalated MoS_2_ was rinsed with DMF and sonicated in PVP/DMF (0.2 mol L^−1^, Mw = 40, 000) for 30 min. The dispersion was centrifuged and washed twice with IPA to remove excessive PVP. Then, the dispersion in IPA was centrifuged at 3000 rpm for 5 min, and the precipitates were discarded. The final dispersion of exfoliated MoS_2_ ink in IPA was ready for further printing.

The synthesis of Ti_3_C_2_T_x_ MXene ink involved selectively etching 1 g of Ti_3_AlC_2_ with an acidic solution of 9 mL deionized water, 3 mL hydrofluoric acid, and 18 mL hydrochloric acid. This mixture was stirred in a high‐density polyethylene container on a heated stir plate for 24 h. Post‐etching, the solution was centrifuged and washed to a neutral pH, followed by delamination using anhydrous lithium chloride. The resulting slurry was further washed, centrifuged, and concentrated into ink, which was then dried in a vacuum oven at 100 °C, resulting in a free‐standing film. Prior to shipping, the ink is treated with argon and sealed in aluminum foil for overnight delivery.

### Fabrication of the Strain‐Isolating Substrate and Sensors

The motion control graphical user interface (GUI) controls the printer's x, y, and z motion stages. Real‐time position and velocity were tracked for x, y, and z during the targeted move and jogging operations. Both aerosol ink flow rates were actively controlled concerning stage position to achieve gradient and voxelated films. For all high‐resolution printing, the inks were primed under sonication for 30 min and then atomized via ultrasonication before being transferred to the printhead. A sheath flow focused the aerosolized ink stream to achieve high printing resolution. Before printing, gradient or homogeneous substrates were treated with plasma to improve surface hydrophilicity. During the AMMP process, a heating stage evaporated the ink solvents to minimize undesired drying effects. Depending on the type of combinatorial materials, additional flash sintering was adopted to achieve the desired microstructures and properties.

All designs of substrates and sensors were printed using AMMP. First, the substrates were designed in AutoCAD, and then the printing paths were automatically generated. To measure the strain distribution, all specimens were prepared with a length of 31 mm, a width of 21 mm, and a gradient composition. The process parameters, including atomizer settings, ink qualities, carrier and sheath gas flow conditions, printer head as well as nozzle geometry, the distance between the nozzle tip and substrate, substrate temperature, etc., are summarized in Tables S5 and S6 (Supporting Information).

### Digital Image Correlation (DIC)

The 2D DIC method was used to characterize the strain distribution and probe Young's modulus distribution of all 5 cases of printed substrates (1D non‐gradient, 1D in‐plane gradient, 1D out‐of‐plane gradient, 2D in‐plane gradient, and 3D gradient). The force‐displacement relation was recorded by uniaxial stretching of the specimen up to failure at a rate of 0.5% (of the initial length) per second via an Instron universal machine (model 5944) with a 50 N load cell. The specimen was mounted in a pair of pneumatic tensile grips, leaving a gauge length of 21 mm. To measure the strain distribution by the DIC method, an ink (Koh‐I‐Noor Rapidraw) was sprayed with an airbrush (Badger, no. 150) to generate high‐quality speckle patterns on the specimen. A whiteboard was used as a background, and a white LED light was shone on the sample during testing to enhance the optical contrast. A Canon ESO 6D DSLR camera recorded changes in the speckle patterns with a Canon 100 mm F/2.8L macro lens at roughly every 2% strain. The resolution of each image was ≈15 µm per pixel, and data was recorded for every four‐pixel length. Images were analyzed by Ncorr40, an open‐source 2D DIC MATLAB software, to obtain the Eulerian strain distributions of the middle region of 11 mm (L) × 11 mm (W). Based on the strain distribution, the modulus distribution was further calculated, the details of which were elaborated in our previous work.^[^ ^51^ ^]^

### Finite Element Analysis (FEA)

The design can be applied to substrates in wearable devices. It was assumed that the sample could be intimately attached to an arbitrary surface, such as the wrist, and stretched when rotating and bending. The sample can deform with the skin without failure. The commercial finite element software ABAQUS was utilized to simulate a gradient substrate's stress and strain distributions under uniaxial and biaxial tension. A 3D model with various modulus distributions designed along the x, y, and z directions was built. The thickness of the uniform soft substrate is 10 µm. On the top of the uniform substrate is a gradient square part with a length of 11 mm and a thickness of 1 µm (Figure S9, Supporting Information). The stress and strain contours in the x‐y plane are shown in Figures S9 and S10 (Supporting Information). By symmetry, only a quarter of the specimen is modeled separately with XSYMM and YSYMM boundary conditions at the left and bottom. An ABAQUS built‐in hyperelastic model with the neo‐Hookean form was assigned to the whole model. The shear moduli for the soft and hard PUD were set as 0.75 to 75 MPa, respectively, and the compressibility parameter to be D = 0 to enforce incompressibility. The sample was stretched in the X direction up to 100% strain for the uniaxial tension case and simultaneously in the X and Y directions up to 100% strain for the biaxial tension case. Element type C3D8H was applied and nonlinear static analysis.

### Nanoindentation

Nanoindentation experiments were conducted using a nanoindenter (Hysitron TriboIndenter, Bruker, Billerica, MA), applying a force of 70 µN to determine the elastic modulus and hardness of the sample material.

### White Light Profilometer

Sample thickness was measured using a white light profilometer (Filmetrics, San Diego, CA) equipped with a 5X magnification objective, which employs chromatic aberration to accurately gauge the vertical displacement between the highest and lowest points of the sample's surface.

### Fluorescent Imaging

Two PUD inks were mixed with different fluorescent dyes and imaged to investigate the gradient composition of printed substrates. Specifically, a red color dye and a green color dye was added to the PUDs to visualize the composition change process better. The fluorescent dyes were purchased from GLO EFFEX with red (UVT‐RD‐1OZ) and green (UVT‐GR‐1OZ). The fluorescent images were captured with a Keyence BZ‐X810 Microscope. RGB analyses quantified the intensity of the two colors on the printed gradient polyurethane films, where the RGB color profiles were obtained by using the ImageJ RGB‐Profiler plugin (National Institutes of Health, USA).

### Resistance Analysis

A custom‐built test setup was used to measure the resistance of electronics under different strains. The straining mechanism consisted of a stepper motor for driving the timing belt, a microcontroller unit to control the precise movement of the timing belt, and a base connected to the timing belt that holds the tested substrate to be stretched. The resistance of the strain gauge is measured using the DAQ system and LabVIEW. The data was continuously monitored and recorded on a computer.

### Flash Sintering

Flash sintering was performed using a Sinteron 2100 system (Xenon Corp., USA) with a 107 mm xenon spiral lamp. The S‐2100 system was configured for maximum pulse durations of 3 ms, with the sintering carried out in an ambient environment. The S‐2100 produced pulse energy (single) ranging from 30 to 2850 J. The detailed flash sintering process can be found from a previous publication.^[^ ^52^ ^]^

### Photodetector Characterization

Photoresponse measurements were conducted using a Keithley 2636A source meter under ambient conditions. A 405 nm laser (Crystal Laser) served as the light source, with power adjustments made via the diode current. Calibration was achieved using an energy meter (PM100D, Thor Labs). Spectrally resolved photocurrents were recorded across wavelengths from 360 to 800 nm at a constant power of 2.5 mW using a supercontinuum broadband laser (SuperK FIANIUM‐NKT photonics) and bandwidth tunable filter (SuperK VARIA‐NKT photonics). Response times were determined by dynamically modulating the laser with a shutter to control the periods of light exposure. The photocurrent mapping measurement can be found from a previous publication.^[^ ^53^ ^]^ The driving voltage of the red and green LEDs was set at 3 V. At the same time, the oxygen saturation was measured by a commercial pulse oximeter (CMS50NA, Contact Medical Systems Co., Ltd.). The oxygen saturation (SpO_2_) can be expressed as a function of the transmitted light ratio (R_OS_) of green and red LEDs. The green and red PPG signal amplitudes were calculated and then calculated the R_OS_.

An informed written consent from all human participants was obtained prior to the research.

## Conflict of Interest

The authors declare no conflict of interest.

## Supporting information



Supporting Information

## Data Availability

The data that support the findings of this study are available in the supplementary material of this article.
