# Supplementary material for: A Printed Microscopic Universal Gradient Interface for Super Stretchable Strain‐Insensitive Bioelectronics
Source: Adv Mater. 2025 Feb 9;37(11):2414203. doi: 10.1002/adma.202414203 (PMC11923513; doi:10.1002/adma.202414203)
Supplement: Supplementary file 1 — Supporting Information [file ADMA-37-2414203-s001.pdf]

ADVANCED MATERIALS

Supporting Information

for *Adv. Mater.*, DOI 10.1002/adma.202414203

A Printed Microscopic Universal Gradient Interface for Super Stretchable Strain-Insensitive Bioelectronics

Kaidong Song, Jingyuan Zhou, Chen Wei, Ashok Ponnuchamy, Md Omarsany Bappy, Yuxuan Liao, Qiang Jiang, Yipu Du, Connor J. Evans, Brian C. Wyatt, Thomas O' Sullivan, Ryan K. Roeder, Babak Anasori, Anthony J. Hoffman, Lihua Jin, Xiangfeng Duan and Yanliang Zhang**

Supplementary Information for:

A Printed Microscopic Universal Gradient Interface for Super Stretchable Strain-Insensitive Bioelectronics

Kaidong Song, Jingyuan Zhou, Chen Wei, Ashok Ponnuchamy, Md Omarsany Bappy, Yuxuan Liao, Qiang Jiang, Yipu Du, Connor J. Evans, Brian C. Wyatt, Thomas O' Sullivan, Ryan K. Roeder, Babak Anasori, Anthony J. Hoffman, Lihua Jin, Xiangfeng Duan and Yanliang Zhang**

K. Song, M. O. Bappy, Y. Liao, Q. Jiang, Y. Du, C. Evans, R. Roeder, Y. Zhang
Department of Aerospace and Mechanical Engineering
University of Notre Dame
Notre Dame, IN 46556, USA
E-mail: yzhang45@nd.edu

J. Zhou, X. Duan
Chemistry and Biochemistry Department
University of California Los Angeles
Los Angeles, CA 90095, USA
E-mail: xduan@chem.ucla.edu

C. Wei, L. Jin
Department of Mechanical and Aerospace Engineering
University of California Los Angeles
Los Angeles, CA 90095, USA

A. Ponnuchamy, T. O'Sullivan, A. Hoffman
Department of Electrical Engineering
University of Notre Dame
Notre Dame, IN 46556, USA

B. Wyatt, B. Anasori
School of Materials Engineering
Purdue University
West Lafayette, IN 47907, USA

Supplementary Note 1. XY-plane printing resolution

Our aerosol-based multi-materials printing (AMMP) approach achieves a high resolution of approximately 20 μm in the X-Y plane. Here are the single filaments printed through soft PUD and hard PUD inks.

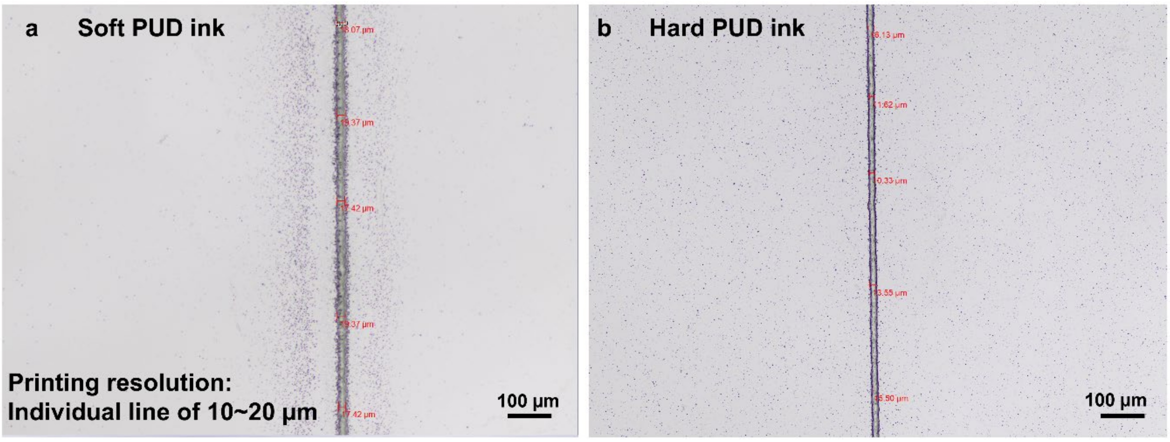

Figure S1. XY-plane printing resolution. Printing of an individual line showing the X-Y plane resolution as small as 10 μm for (a) soft PUD, and (b) hard PUD inks (Nozzle size: 30 gauge).

Supplementary Note 2. Z-plane printing resolution

Figure S2a shows the 3D scan of the printed 1-layer PUD film by using a white light profilometer (Filmetrics, Profilm3D). White light interferometry technology is used by this kind of profilometer to provide quantitative surface topological information. It is a nondestructive, non-contact type of measurement method offering good technology to measure film thickness. The film thickness shown in **Figure S2b** demonstrates the deposition resolution along Z direction as small as around 500 nm.

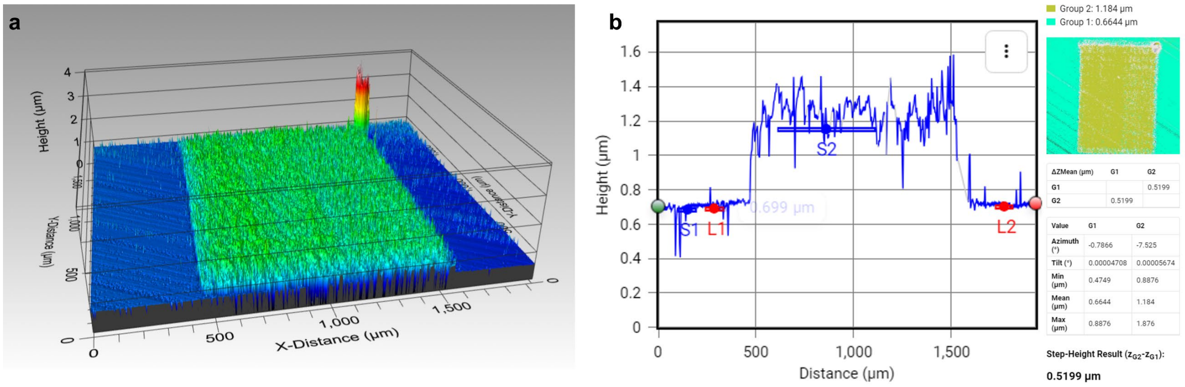

Figure S2. Z-plane printing resolution. (a) A typical white light profilometer scan across the printed 1-layer PUD film shows the deposition resolution as small as 500 nm (nozzle size: 25 gauge). (b) Surface topology of the printed film.

Supplementary Note 3. Design of each gradient pattern

Figure S3 illustrates the dimensions of various Universal Gradient Interface (UGI) designs. The non-gradient design, used as a reference, features an abrupt modulus transition along the X-direction. The 1D in-plane gradient design exhibits a gradual modulus change in the X-direction. Conversely, the 1D out-of-plane gradient design transitions modulus changes along the Z-direction. The 2D in-plane gradient design modifies modulus progressively in both the X and Y directions. Lastly, the 3D gradient design systematically alters the modulus across all three dimensions: X, Y, and Z.

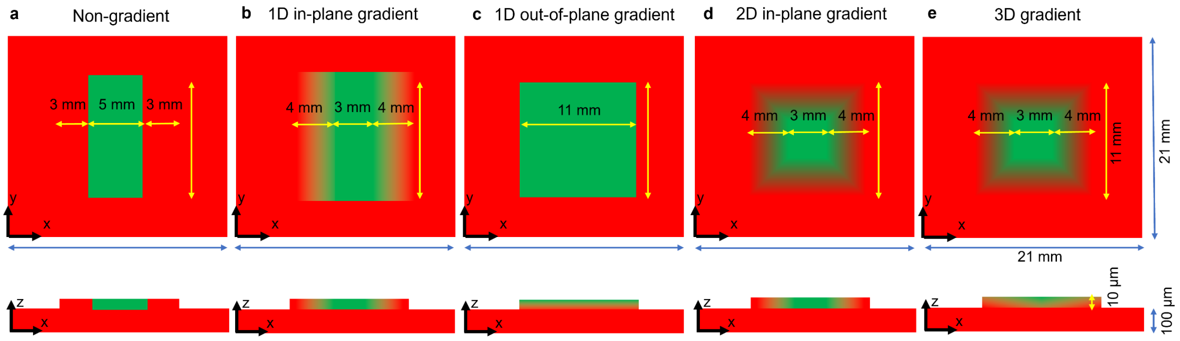

Figure S3. Design of each non-gradient and gradient pattern.

Supplementary Note 4. Non-gradient and 1D out-of-plane UGI properties

Figures S4a and **S4d** show the printed non-gradient and 1D out-of-plane interface patterns which are used for comparison purposes. **Figures S4b** and **S4e** show the site-specific component ratio of the non-gradient and 1D out-of-plane gradient design. The top surface principal Lagrange strain map obtained from DIC for non-gradient and 1D out-of-plane gradient samples under 100% global uniaxial external stretch (**Figures S4c** and **S4f**) illustrates the non-gradient and 1D out-of-plane gradient samples already break at the soft-stiff interfaces.

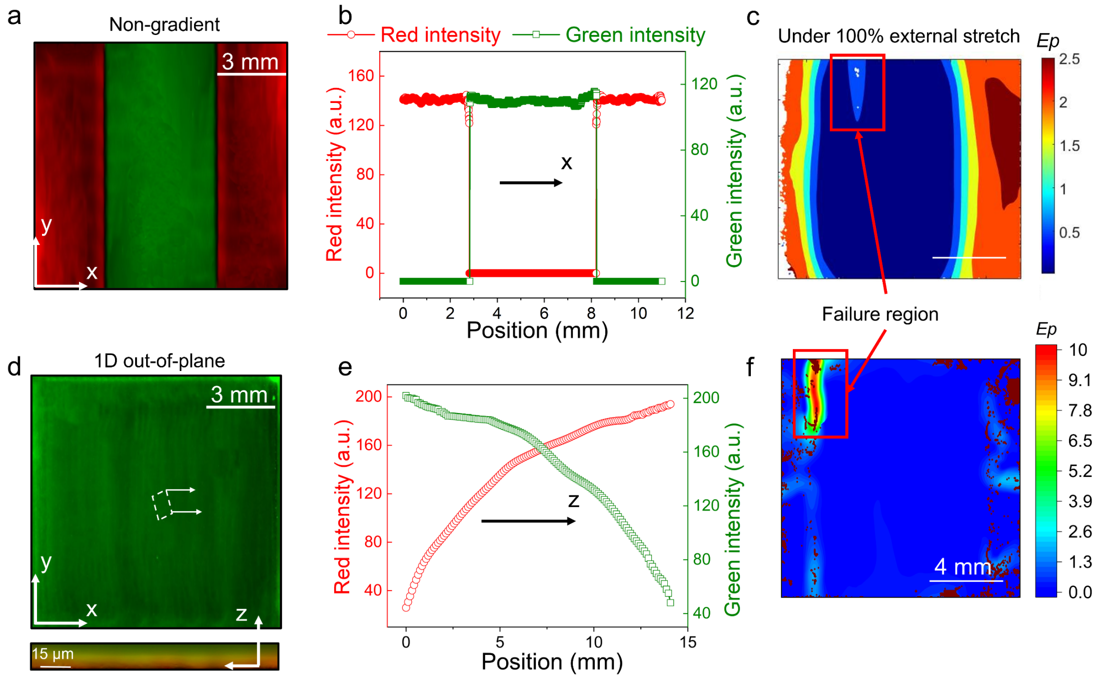

Figure S4. Non-gradient and 1D out-of-plane UGI properties. (a) Printed pattern, (b) site-specific component ratio, and (c) DIC under 100% uniaxial stretch ratio for non-gradient design. (d) Printed pattern, (e) site-specific component ratio, and (f) DIC under 100% uniaxial stretch ratio for 1D out-of-plane gradient design.

Supplementary Note 5. DIC figures for each design at crack

Figure S5 shows the top surface maximum principal logarithmic strain map obtained from DIC for each design when a crack occurs.

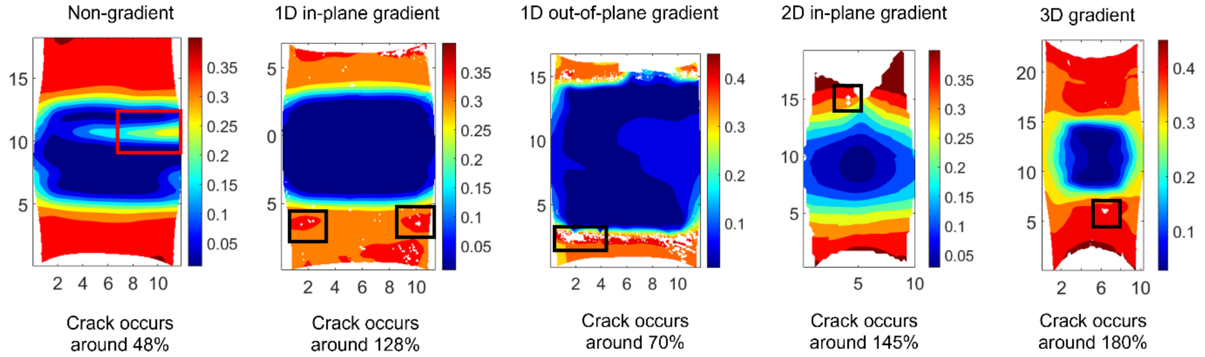

Figure S5. DIC figures for each design at crack.

Supplementary Note 6. Finite Element Analysis (FEA) results under uniaxial tension

Figure S6 illustrates the maximum principal stress and logarithmic strain distributions obtained from FEA under 100% uniaxial tension for each design. In non-gradient designs, the abrupt modulus transition at soft-stiff interfaces leads to significant strain mismatches and elevated stress levels, particularly at the corners. Similarly, in 1D out-of-plane designs, stress and strain concentrations occur at the corners of the stiff region due to the absence of gradient transitions along the stretch direction. In contrast, UGI designs (1D in-plane, 2D in-plane, and 3D gradients) effectively mitigate stress and strain concentrations by providing smoother modulus transitions. These transitions reduce localized stress, allowing a broader region to distribute it more evenly. Additionally, the gradient designs relocate high-stress regions to the stiff (hard PU) areas, significantly enhancing global failure resistance. The strain progressively increases along the gradient as softer regions deform more under the same external loads. Among these, the 3D gradient design achieves the smoothest strain distribution, further optimizing stress distribution and minimizing strain concentrations.

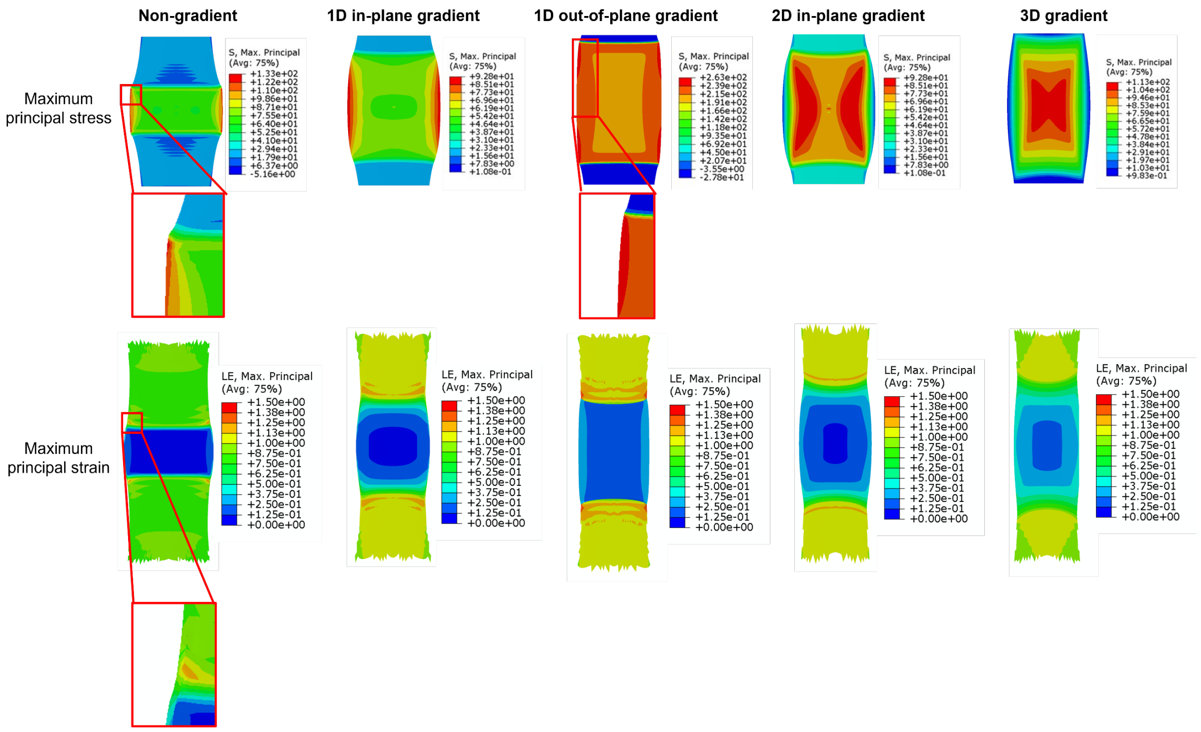

Figure S6. FEA results under uniaxial tension.

Supplementary Note 7. Stretching process for each design

Figure S7 shows the optical images of the stretching process of 0-100% uniaxial stretch ratio and the images of cracks occurring in each UGI.

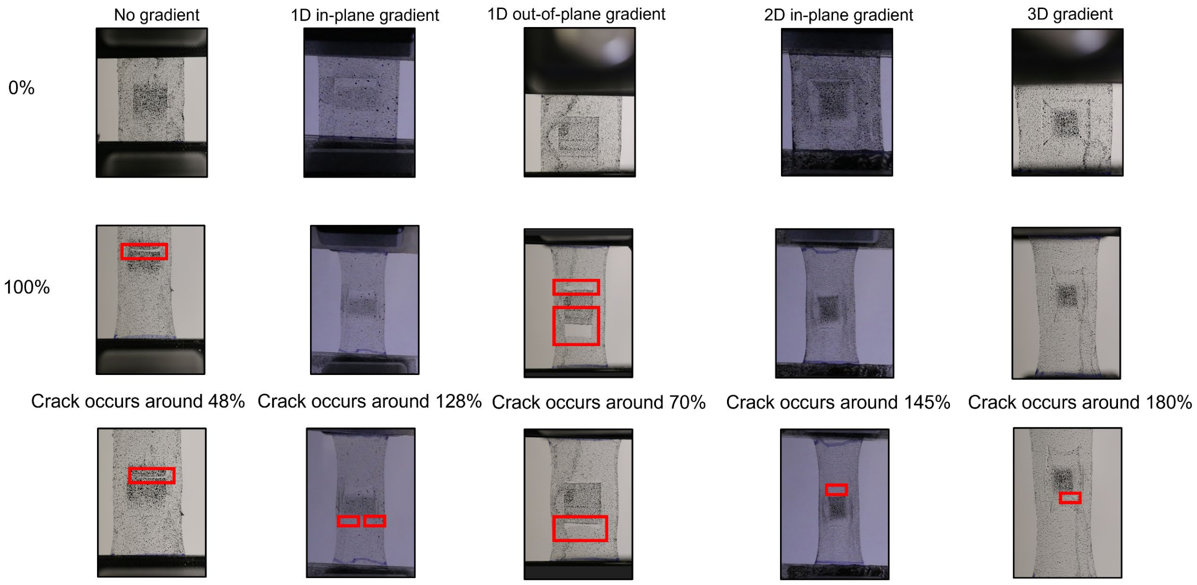

Figure S7. Optical image of stretching process for each design.

Supplementary Note 8. Tensile test for non-gradient and 3D UGI design

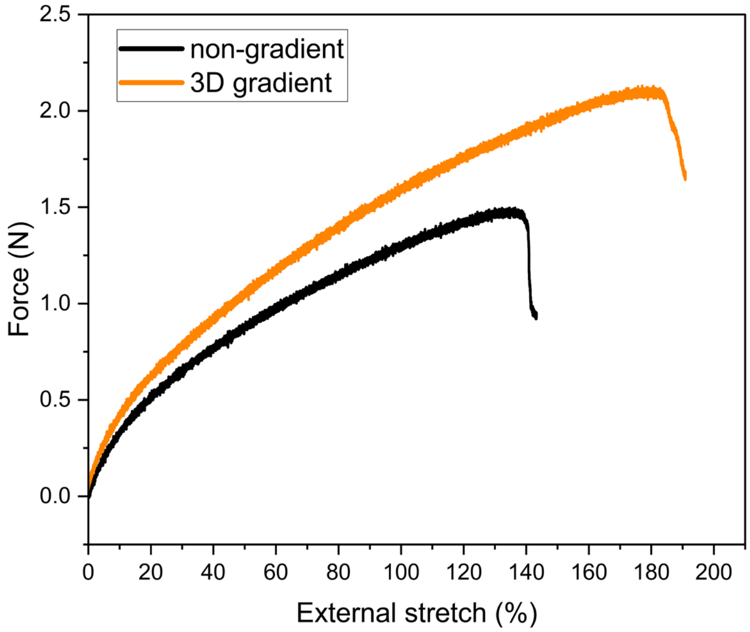

Figure S8. Tensile test for non-gradient and 3D UGI design.

Supplementary Note 9. Simulation for each design for uniaxial stretch ratio of 100%

Figure S9 shows the simulation results of maximum principal strain and principal maximum stress under a uniaxial stretch ratio of 100% for each UGI. **Figures S9a** and **S9b** show the schematic and dimensions of the simulation process. By symmetry, only a quarter of the specimen is modeled with XSYMM and YSYMM boundary conditions at the left and bottom separately. **Figures. S9c** to **S9g** illustrate the maximum principal logarithmic strain distribution of each design. **Figures. S9h** to **S9l** show the principal maximum stress distribution of each design.

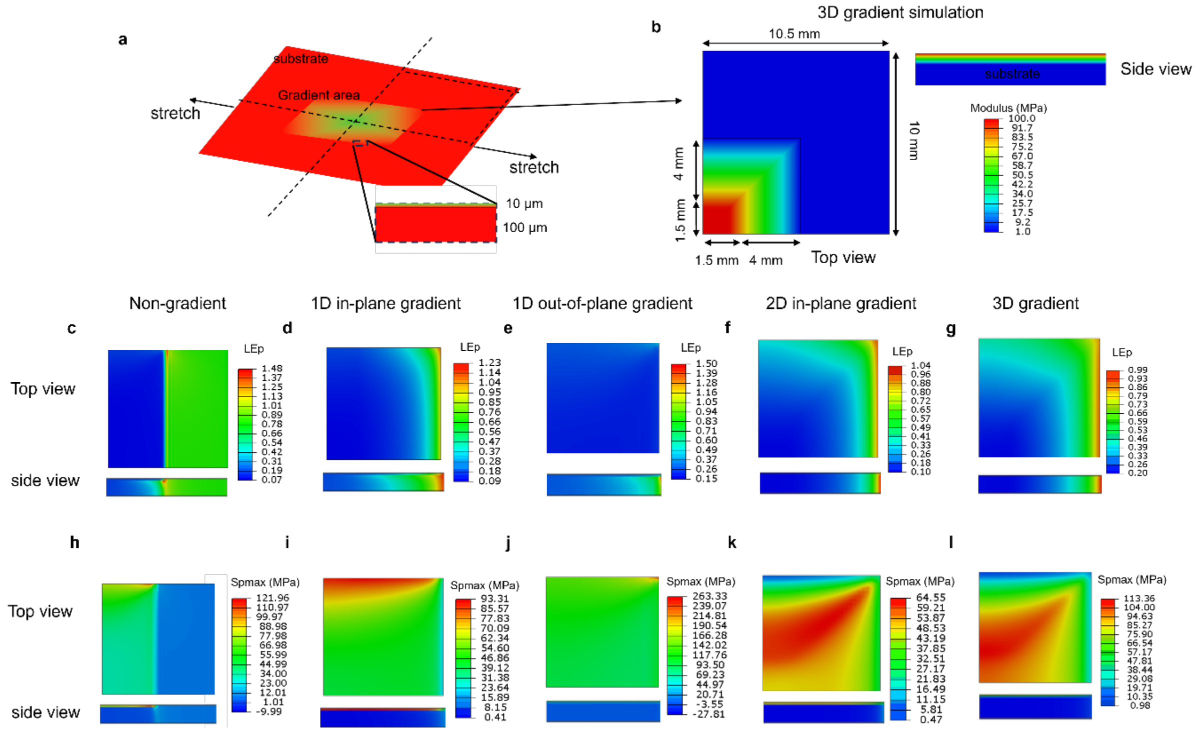

Figure S9. Simulation for each design for uniaxial stretch ratio of 100%. (a) Schematic of the simulation setup. (b) Simulation dimensions with 2D in-plane gradient design as an example. (c)-(g), Principal logarithmic strain distribution of each design. (h)-(l), Maximum principal stress distribution of each design.

Supplementary Note 10. Simulation for each design for bi-axial stretch ratio of 100%

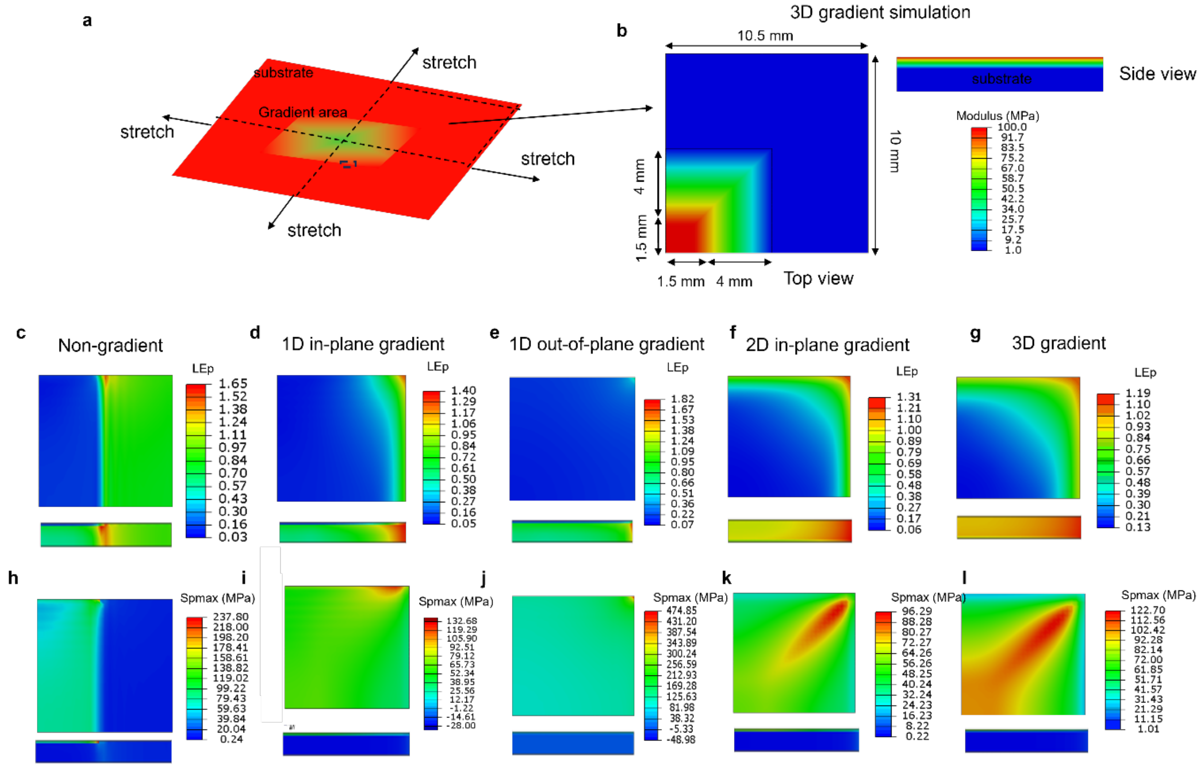

Figure S10. Simulation for each design for bi-axial stretch ratio of 100%. (a) Schematic of the simulation setup. (b) Simulation dimensions with 3D in-plane gradient design as an example. (c)-(g), Principal logarithmic strain distribution of each design. (h)-(l), Maximum principal stress distribution of each design.

Supplementary Note 11. Flash sintering of printed Au sensor

Flash sintering is one type of photonic sintering process where white flashlight energy is utilized to create local heating, resulting in the evaporation of solvents, integration of the nanoparticles being sintered, increasing the electrical and thermal conductivity, reducing porosity, etc. In this study, we used a Sinteron 2100 (Xenon Corporation, USA) integrated with a 107 mm Xenon spiral flash lamp. The xenon flash lamp generates white light by converting the electrical energy into light energy.[1] The short-pulsed white light from the xenon lamp covers a small part of UV light (380 nm), infrared light (950 nm), and the entire length of visible light.[2] Due to the surface plasmon resonance effect of the metal nanoparticles in the range of the visible light spectrum, when flash sinters metal nanoparticles, they absorb the light energy, resulting in local heating and completing the sintering process.[1] As the whole process is completed in a few seconds, the flash sintering is faster than other sintering processes, such as thermal sintering, laser sintering, etc. Targeted and localized sintering is also possible by flash sintering.

Here, the printed gold nanoparticles were sintered in air at 2 KV flash lamp voltage, 800 µs flashlight duration, and 3 consecutive pulses with a pulse delay of 858 ms, as shown in **Figure S11a**. As the optimized sintering time is less than 2s and the PUD substrate only absorbs the UV light of the xenon flash lamp,[1] metal nanoparticles can be sintered on a PUD substrate without potentially damaging it. The comparison of the printed gold filaments before and after flash sintering are illustrated in **Figures S11b** and **S11c**.

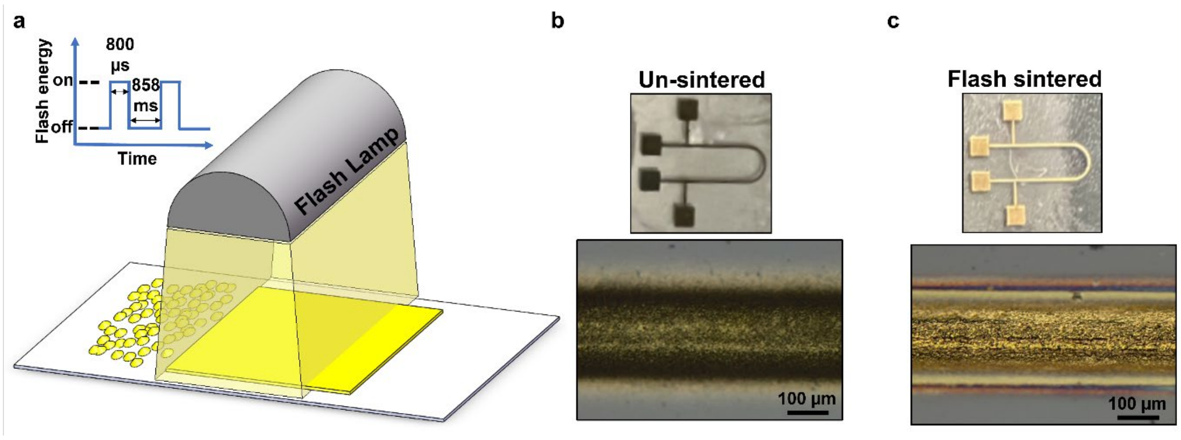

Figure S11. Flash sintering of printed Au sensor. (a) ultra-fast flash sintering of the printed gold and the sintering condition. Printed gold (b) before and (c) after flash sintering.

Supplementary Note 12. Stability curve of the printed MXene-based sensor

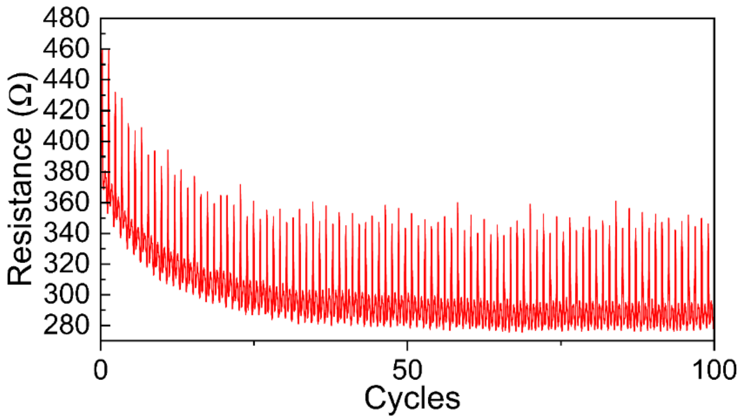

Figure S12. The stability curve of the printed MXene-based sensor on soft PUD substrate is under 20% uniaxial strain for 100 cycles.

Supplementary Note 13. 3D UGI cycling test

To validate the mechanical repeatability of the UGI and its ability to maintain stable electrical properties in integrated devices, we conducted a 1,000-cycle tensile test at a 50% stretch ratio using a gold-based sensor on a 3D gradient UGI (**Figure S13a**). The resistance change of the sensor was recorded, revealing a low and repeatable relative resistance change (RRC) ($\Delta R/R_0$) of less than 1.6% throughout the cycles (**Figure S13b**). These results highlight the durability and resilience of the 3D UGI under cyclic loading conditions.

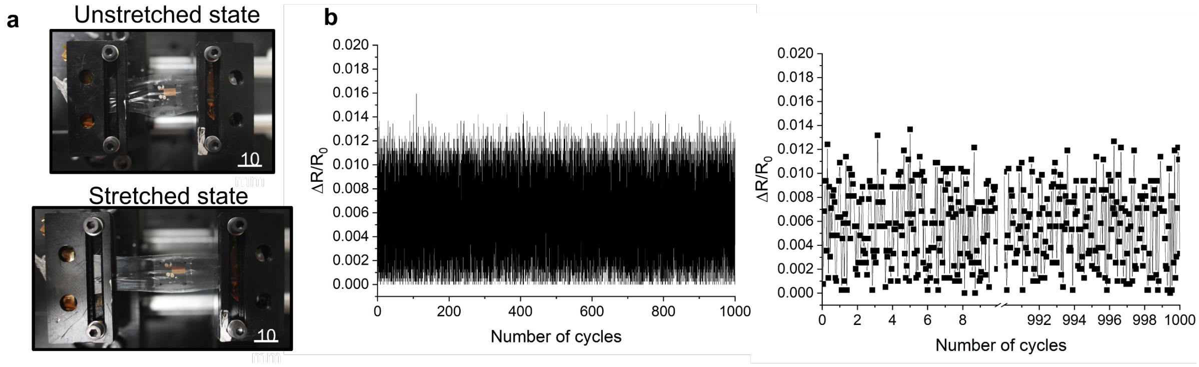

Figure S13. (a) Pictures of UGI under unstretched and stretched states. (b) RRC vs. stretch ratio for gold-based sensor on a 3D gradient UGI.

Supplementary Note 14. Au-based temperature sensor

Here the detailed design and fabrication process of the stretchable temperature sensor are illustrated in **Figure S14a**. **Figure S14b** shows the resistance change of the temperature sensor accurately following the changed temperature, which demonstrates the accuracy of the temperature sensor.

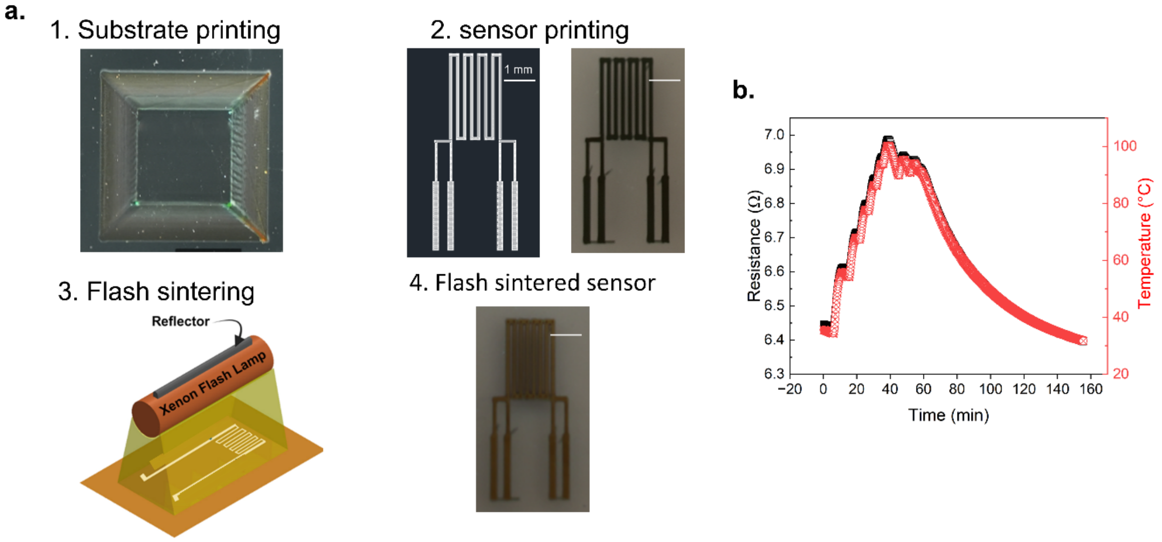

Figure S14. (a) The design and fabrication process of the temperature sensor. (b) The curve of resistance of the sensor under different temperatures.

Supplementary Note 15. Humidity test

To further evaluate the UGI's performance under varying environmental conditions, we tested its robustness at different humidity levels. Here we used a humidifier to control the environmental humidity (**Figure S15a**). The sensor printed on the 3D gradient UGI showed consistent performance with minimal RRC change (< 1.3%) during stretching (50% stretch ratio) under different humidity conditions, confirming the platform's reliability (**Figure S15b**).

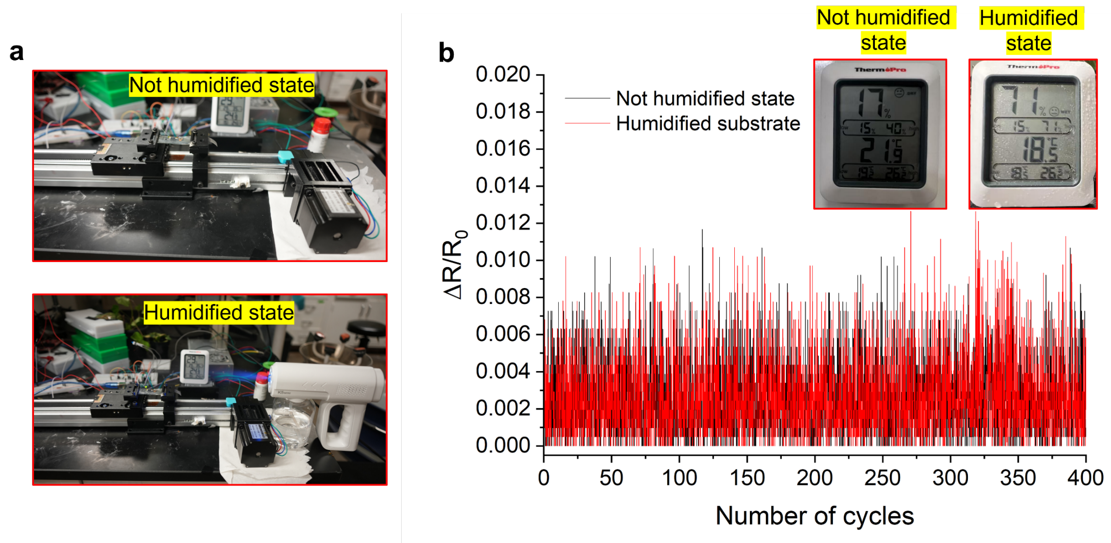

Figure S15. (a) Pictures of UGI under not humidified and humidified states. (b) RRC vs. stretch ratio for printed sensors under different humidified states.

Supplementary Note 16. Substrate transparency analysis

Here the transparency of PUD substrate under different wavelengths is illustrated in **Figure S16**. The high transparency confirms its capability for optical sensor applications.

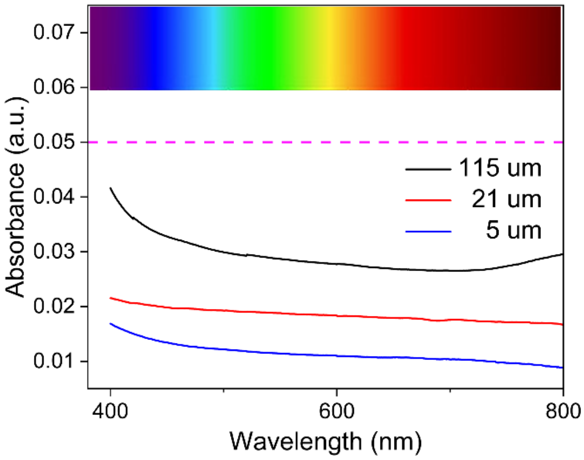

Figure S16. Substrate transparency analysis.

Supplementary Note 17. Optical sensor fabrication process

The MoS_2-based optical sensor or photodetector fabrication process is illustrated here. First, the MoS_2 was printed on a gradient substrate. Then, the MoS_2 layer was sintered in air at 2.4 KV flash lamp voltage, 800 µs flashlight duration, and 2 consecutive pulses with a pulse delay of 1360 ms through flashlight sintering. At last, MXene was printed on MoS_2 as electrodes.

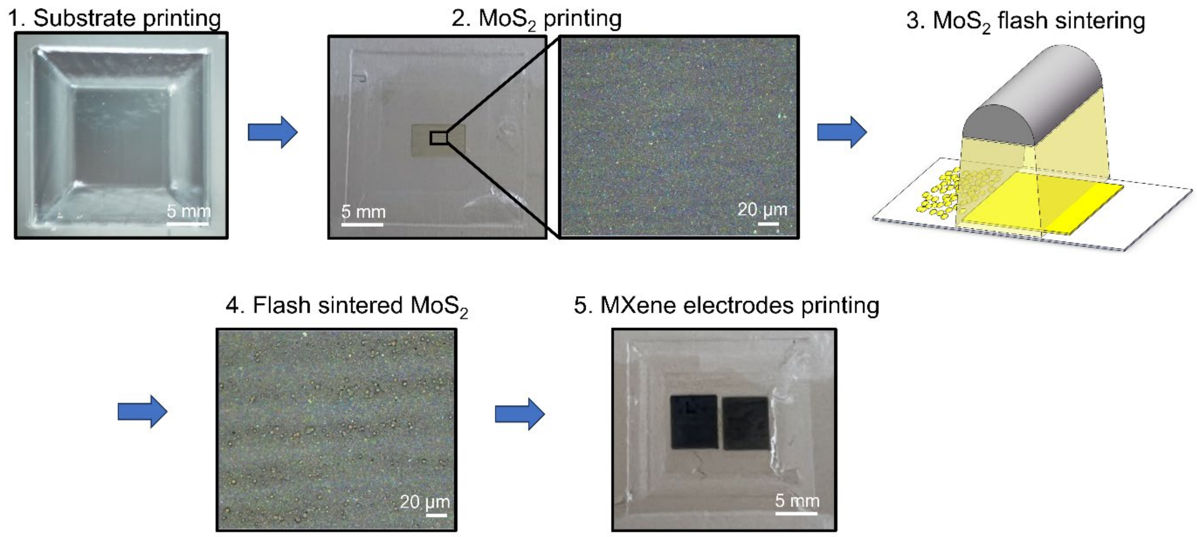

Figure S17. Optical sensor fabrication process.

Supplementary Note 18. Responsivity of printed optical sensor

For measuring the photodetector responsivity, we can assume that the intensity is uniform throughout the device channel since the laser spot size is substantially larger than the channel and covers the entire device. The total power was determined by multiplying the channel area by the intensity at the set laser current. The effective channel area is approximately 300 µm x 300 µm, and the input 405 nm laser has a diameter of 460 µm. Thus, we can calculate responsivity, $R = I_{pc}/P$, where I_{pc} is the photocurrent and P is the laser input power. The high responsivity observed is primarily due to the applied voltage across the photodetector, enhancing the electric field, which in turn affects charge carrier dynamics. This applied voltage improves photocurrent generation by decreasing carrier recombination and boosting light-to-electricity conversion efficiency. In MoS_2 photoresistors, which operate based on the photoconductive principle, such voltage application not only increases conductivity with light exposure but also facilitates charge separation, further amplifying the photocurrent and thereby enhancing responsivity.

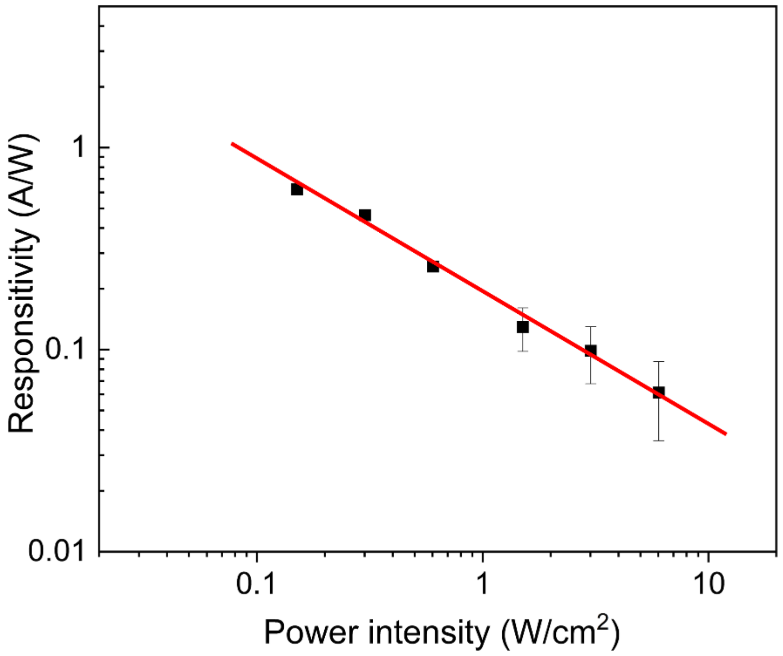

Figure S18. Responsivity as a function of illumination power measured with a 405 nm laser under a bias voltage of 5V.

Supplementary Note 19. Response time and responsivity relationship of printed optical sensor

Figure S19a shows the relationship between the printed MoS_2 thickness and response time. A thinner layer of MoS_2 in photodetectors generally leads to a faster response primarily due to the reduced volume of material through which charge carriers need to move. While too thinner MoS_2 layers might not absorb light as effectively due to their reduced thickness, which can decrease the generation rate of electron-hole pairs. This reduced generation rate means fewer carriers are available to contribute to the photocurrent, potentially increasing the time it takes to reach a detectable signal level. **Figure S19b** shows the comparison of responsivity and response time in the devices in this work with previously reported all-printed photodetectors in the visible light range.

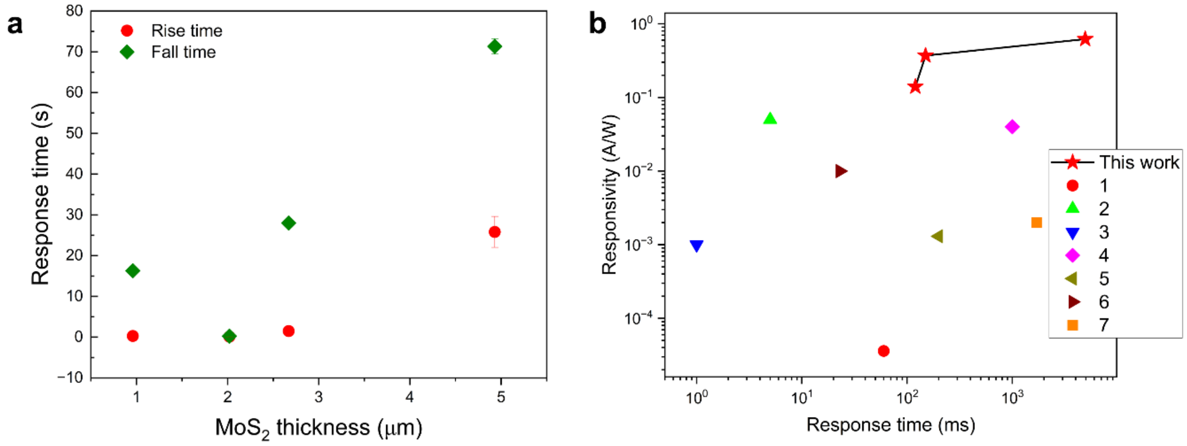

Figure S19. (a) Detailed response time and responsivity relationship. (b) Comparison of responsivity and response time in the devices in this work with previously reported all-printed photodetectors in the visible light range.

Supplementary Note 20. Setup for optical sensor array application

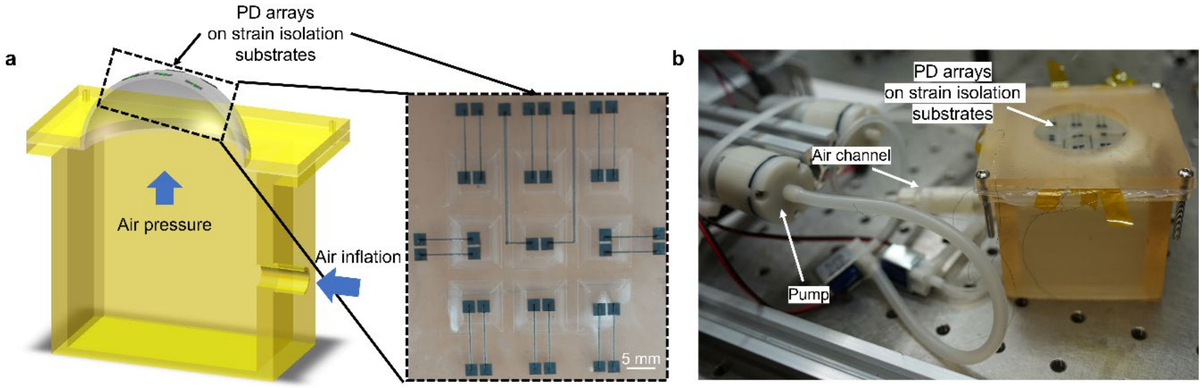

Figure S20. (a) Schematic illustration of the cross-sectional view of the pressure-tunable cylindrical cavity. (b) Optical image of the pressure-tunable cylindrical cavity connected with a mini pump.

Supplementary Note 21. Simulation of curved surface deformation

Figure S21a illustrates the dimensions of the surface being simulated. Owing to symmetry, only one-quarter of the specimen is modeled, as depicted in **Figure S21b**. **Figure S21c** presents the simulated results of the strain and stress distributions.

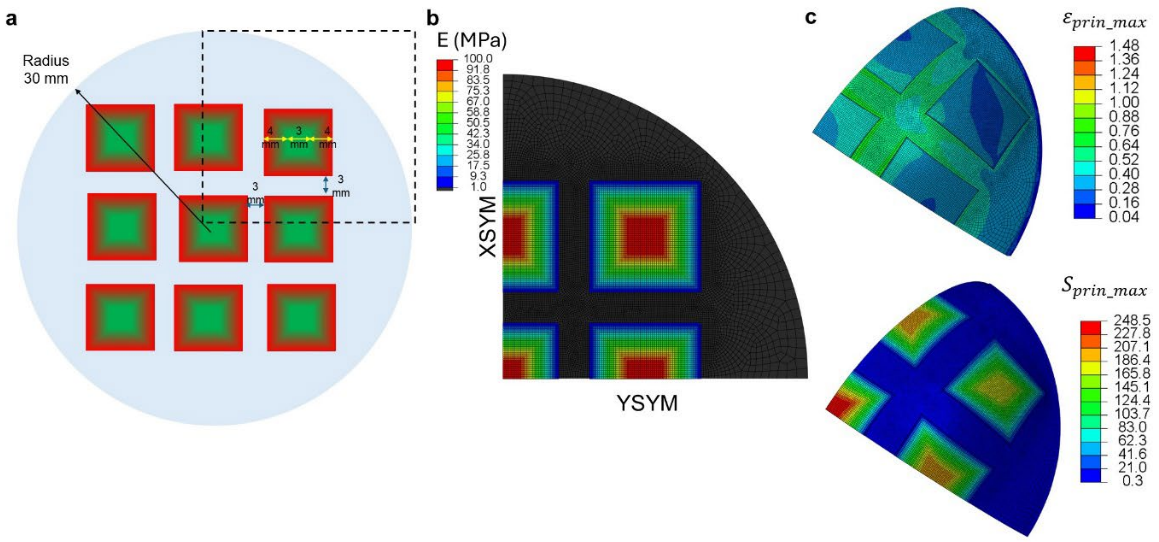

Figure S21. (a) Schematic illustration of dimension for simulation. (b) Modulus distribution of the sample. (c) Strain and stress distribution during stretching.

Supplementary Note 22. 3D photodetector array location coding and photocurrent

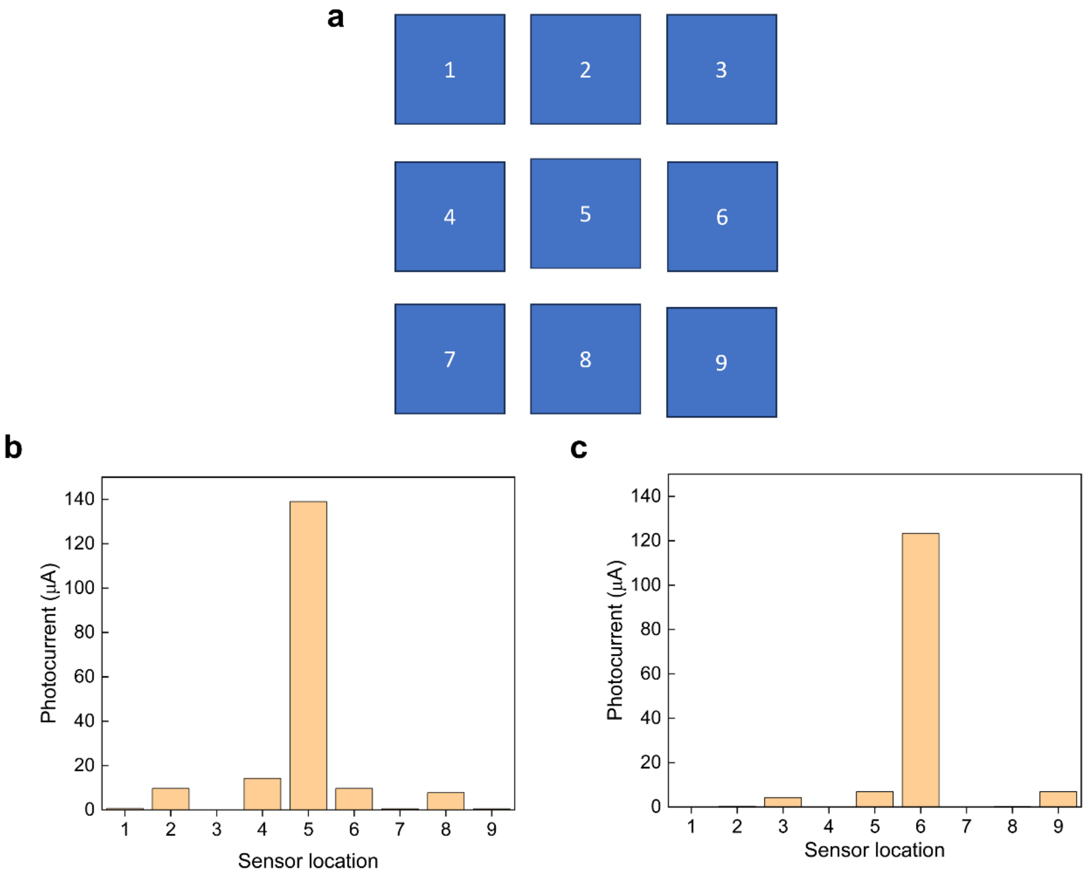

Figure S22. (a) 3D photodetector array location coding. Photocurrent of printed 3D photodetector array (b) under normally-incident illumination and (c) under obliquely incident light.

Supplementary Note 23. Electrical connection of the demonstrators

Figure S23 illustrates the connection setup for the skin-mounted sensors to the external measurement equipment during the experiments. Flexible conductive leads (36 AWG tinned copper wires) and adhesive electrodes (silver conductive paint) were utilized to ensure reliable and robust measurements while prioritizing user comfort. To secure the system, a thin layer of transparent tattoo paper was applied over the device, sealing it completely.

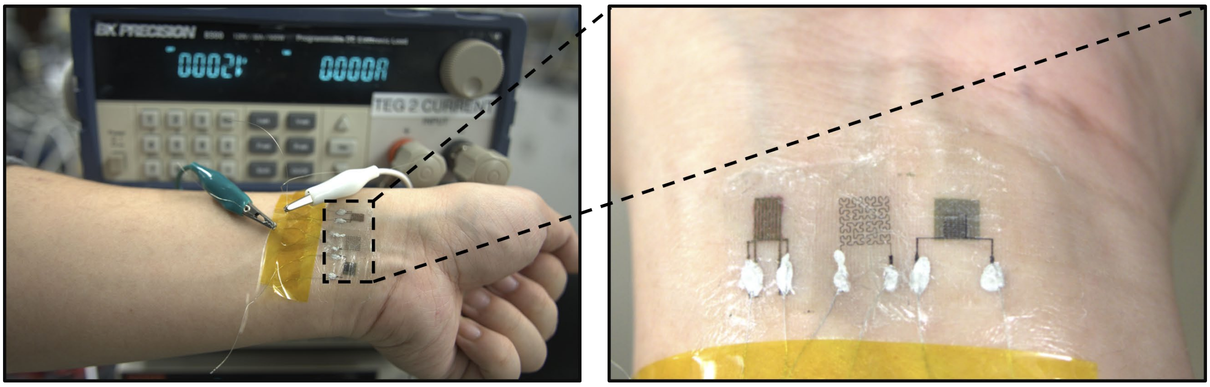

Figure S23. Connection setup for the skin-mounted sensors to the external measurement equipment.

Supplementary Note 24. Oximeter calculation

Blood oxygen saturation (SO_2) is quantified according to **Equation S1**. Here, C_{HbO2} and C_{Hb} are the concentrations of oxy-hemoglobin and deoxy-hemoglobin, respectively.

$$SO_2 = \frac{C_{HBO_2}}{C_{HBO_2}+C_{HB}} \quad \text{(S1)}$$

In this context, we use the characteristic value "Ros", the ratio of signal from red (A_{rd}) and green (A_{gr}) light according to Beer–Lambert's law (shown in **Equation S2**) to represent the blood oxygen saturation (SO_2):

$$R_{os} = \frac{A_{rd}}{A_{gr}} \approx {\frac{AC_{rd}}{DC_{rd}}}\Big/{\frac{AC_{gr}}{DC_{gr}}} \quad \text{(S2)}$$

AC_{rd} and DC_{rd}, along with AC_{gr} and DC_{gr}, represent signals derived from red (650 nm) and green (530 nm) light PPG waveforms, respectively. As demonstrated in **Figures S24a**, these signals can be categorized into DC and AC components when converting light signals to electrical signals. The DC component is relatively constant across non-arterial tissues like muscles, bones, veins, and connective tissues, provided there is minimal movement at the measurement site. In contrast, the AC component varies due to dynamic changes in arterial light absorption, capturing fluctuations in arterial blood volume linked to cardiac cycles, specifically the phases of diastole and systole.

Direct comparison of red and green light signals may be unreliable due to variations in their light sources. The disparity in the DC component stems from the differing absorption rates of red and green light by skin tissue. To ensure equitable analysis of PPG signals from both light sources, the DC components are typically normalized through proportional calculations, as depicted in **Figures. S24b** and **S24c**. This normalization facilitates straightforward calculation of the R_{os} value:

$$R_{os} \approx \frac{AC_{rd}}{AC_{gr}} \quad \text{(S3)}$$

For illustration, we make the measurement during a breath hold process, where SO_2 levels affect the oximeter readings. At an SO_2 of 99%, the AC_{rd} is 0.135 μA, and AC_{gr} is 0.150 μA, yielding a R_{os} value of 0.9. When SO_2 drops to 96%, AC_{rd} increases to 0.178 μA while AC_{gr} decreases to 0.134 μA, resulting in a R_{os} of 1.328. This demonstrates a significant rise in R_{os} correlating with decreases in SO_2 during breath hold process. Consequently, our oximeter effectively measures and interprets real-time, non-invasive oxygen saturation, facilitating ongoing, discreet surveillance of physiological changes for early anomaly detection and disease prevention.

According to the Beer-Lambert Law and prior research,[3,4] SO_2 is determined by **Equation S4**, which directly correlates SO_2 with the R_{os} value. The terms $\varepsilon_{rd,Hb}$ and $\varepsilon_{gr,Hb}$ represent the molar absorptivities of deoxy-hemoglobin at the red (λ = 626 nm) and green (λ = 530 nm)

wavelengths, respectively. In a similar vein, $\varepsilon_{rd;HbO2}$ and $\varepsilon_{gr;HbO2}$ denote the molar absorptivities of oxy-hemoglobin at these same red and green wavelengths, respectively.

$$SO_2(R_{os}) = \frac{\varepsilon_{rd,Hb} - \varepsilon_{gr,Hb} R_{os}}{(\varepsilon_{rd,Hb} - \varepsilon_{rd,HbO_2}) + (\varepsilon_{gr,Hb} - \varepsilon_{gr,HbO_2}) R_{os}} \quad \text{(S4)}$$

To address the limitations of the Beer-Lambert Law, empirical corrections are necessary. By aligning the collected R_{os} values with the SO_2 values from a commercial sensor, a calibration curve is presented in **Figure S24d**.

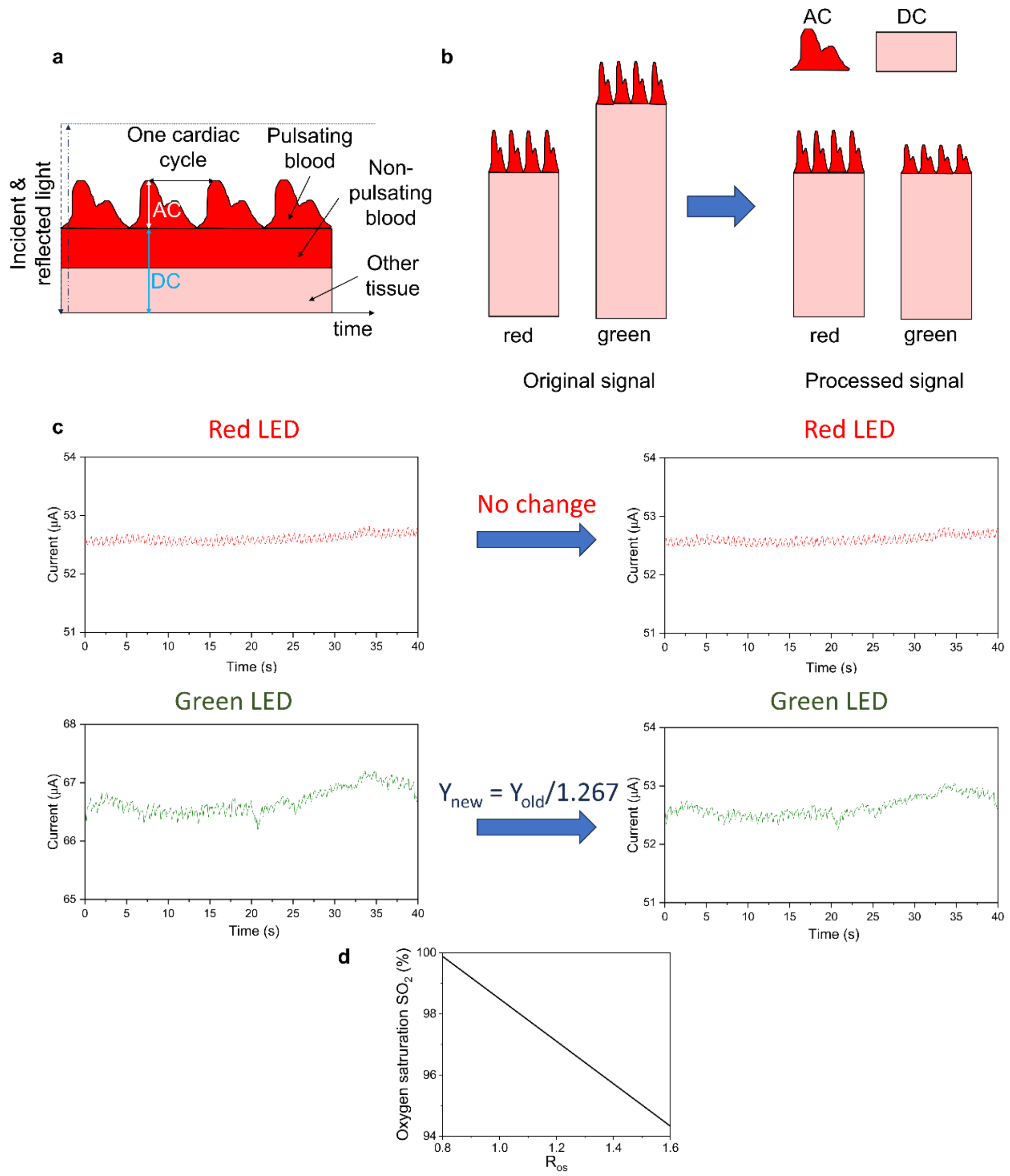

Figure S24. (a) Composition of AC and DC components in the PPG waveform. (b) Schematic representation of both original and processed signals for red (650 nm) and green (530 nm) light.

(c) Processing diagram used to mitigate the influence of DC components. (d) Graph of matching R_{os} values with SO_2.

Supplementary Note 25. On skin performance

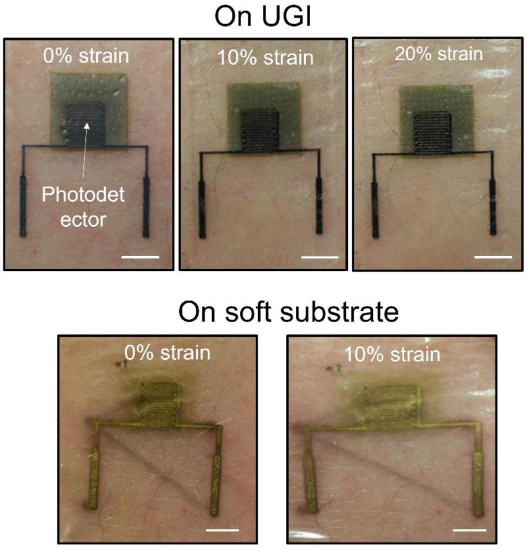

Figure S25. Oximeter printed on UGI and soft substrate before and after on-skin uniaxial stretching. Scale bar, 2.5 mm.

Table S1. Typical stretchable materials and their performance.

Ref	Plot number in main content	Resistance increase	Methods
[5]	9	The resistance of the elastic conductors remained the same under high bending strain and increased slightly (≈ 14%) at a tensile strain of 100%	Apply pre-strain
[6]	27	Larger pre-strain (20, 30, 40%) resulted in stable conductance	Apply pre-strain
[7]	31	Stable of stretching at $\varepsilon = 0.7$	Intrinsically stretchable material
[8]	32	Resistance maintained at 60% (longitudinal), 140% (transverse) strain	Intrinsically stretchable material
[9]	33	The resistance keeps increasing slightly	Intrinsically stretchable material
[10]	34	Conductivity maintained up to 50% strain	Wrinkle and Serpentine
[11]	35	$\Delta R/R0 = 0.1$ at 80% strain	Wrinkle
[12]	36	Conductivity maintained up to 50% strain	Serpentine
[13]	37	Resistance slightly increased up to 200% strain	Serpentine
[14]	38	Conductivity maintained up to 100% strain	Serpentine
[15]	39	Resistance slightly increased up to 200% strain	Rigid island
[16]	40	The resistance keeps increasing slightly	Gradient substrate
[17]	41	The resistance keeps increasing slightly	Rigid island

Table S2. Literature comparison for Fig. S16b.

Ref	Plot label number	Printing method	Response time (ms)	Responsivity (A/W)
	This work	AJP	120	0.14
	This work	AJP	150	0.37
	This work	AJP	4930	0.62
[18]	1	Inkjet	60	3.6×10^{-5}
[19]	2	Inkjet	5	0.05
[20]	3	Inkjet	1	0.001
[21]	4	Electrohydrodynamic	1000	0.004
[22]	5	Laser printing	200	0.0013
[23]	6	Inkjet	23	0.01
[24]	7	Inkjet	1700	0.002

Table S3. Typical motion artifact-free devices' performance.

Ref	Sensor type	Percent signal variance/drift	Applied strain
This work	Oximeter	<1.7%	≈ 20%
[25]	Hydration sensor	≈ 14.7%	≈ 10%
[26]	Epidermal antenna	≈ 80%	≈ 20%
[27]	EMG electrode array	≈ 60%	Unknown
[28]	Solid-state analyte sensor	≈ 21%	Unknown
[29]	Pulse sensor	≈ 50%	Unknown

Table S4. Typical ink formulation of nanoparticle inks.

Materials	Solvent	Co-solvent	Mass percentage	Surfactants/Additives
Soft PUD	Water	DMSO	30%	EG
Hard PUD	Water		27.3%	EG
PEDOT: PSS	Water		0.8%	
Gold	Xylene		2%	
AgNWs	Water		1%	
MXene	Water		0.8%	
MoS_2	IPA		0.6%	Terpinol

Table S5. Aerosol jet printing parameters for each gradient substrate design.

Parameters	Non-gradient	Linear gradient	Z-gradient	XY-gradient	XYZ-gradient
Nozzle nominal I.D. (μm)	410	410	410	410	410
Sheath gas flow rate (sccm)	60	60	60	60	60
Soft PUD ink flow rate (sccm)	30	10-30	10-30	10-30	10-30
Hard PUD ink flow rate (sccm)	30	17-30	17-30	17-30	17-30
Platen temperature (°C)	50	50	50	50	50
Print speed (mm/s)	2	2	2	2	2
Ultrasonic atomizing voltage (V) (soft)	43	43	43	43	43
Ultrasonic atomizing voltage (V)(hard)	43	43	43	43	43

Table S6. Aerosol jet printing parameters for each sensor.

Parameters	Strain sensor	Temperature sensor	Oximeter (channel)	Oximeter (electrode)
Nozzle nominal I.D. (μm)	250	250	410	250
Sheath gas flow rate (sccm)	80	80	60	80
Ink flow rate (sccm)	10	10	15	12
Platen temperature (°C)	50	50	50	50
Print speed (mm/s)	2	2	2	2
Ultrasonic atomizing voltage (V)	43	43	43	43

References

[1] G. L. Goh, H. Zhang, T. H. Chong, W. Y. Yeong, *Adv Electron Mater* **2021**, *7*, 1.

[2] Y. R. Jang, S. J. Joo, J. H. Chu, H. J. Uhm, J. W. Park, C. H. Ryu, M. H. Yu, H. S. Kim, *A Review on Intense Pulsed Light Sintering Technologies for Conductive Electrodes in Printed Electronics*, Korean Society For Precision Engineering, **2021**.

[3] C. M. Lochner, Y. Khan, A. Pierre, A. C. Arias, *Nat Commun* **2014**, *5*, 1.

[4] H. Lee, W. Lee, H. Lee, S. Kim, M. V. Alban, J. Song, T. Kim, S. Lee, S. Yoo, *ACS Photonics* **2021**, *8*, 3564.

[5] K. H. Kim, M. Vural, M. F. Islam, *Advanced Materials* **2011**, *23*, 2865.

[6] A. Miyamoto, S. Lee, N. F. Cooray, S. Lee, M. Mori, N. Matsuhisa, H. Jin, L. Yoda, T. Yokota, A. Itoh, M. Sekino, H. Kawasaki, T. Ebihara, M. Amagai, T. Someya, *Nat Nanotechnol* **2017**, *12*, 907.

[7] M. Shin, J. H. Song, G. H. Lim, B. Lim, J. J. Park, U. Jeong, *Advanced Materials* **2014**, *26*, 3706.

[8] G. D. Moon, G. H. Lim, J. H. Song, M. Shin, T. Yu, B. Lim, U. Jeong, *Advanced Materials* **2013**, *25*, 2707.

[9] P. Lee, J. Lee, H. Lee, J. Yeo, S. Hong, K. H. Nam, D. Lee, S. S. Lee, S. H. Ko, *Advanced Materials* **2012**, *24*, 3326.

[10] D. C. Hyun, M. Park, C. Park, B. Kim, Y. Xia, J. H. Hur, J. M. Kim, J. J. Park, U. Jeong, *Advanced Materials* **2011**, *23*, 2946.

[11] V. R. Feig, H. Tran, M. Lee, Z. Bao, *Nat Commun* **2018**, *9*, 1.

[12] J. Park, S. Choi, A. H. Janardhan, S. Y. Lee, S. Raut, J. Soares, K. Shin, S. Yang, C. Lee, K. W. Kang, H. R. Cho, S. J. Kim, P. Seo, W. Hyun, S. Jung, H. J. Lee, N. Lee, S. H. Choi, M. Sacks, N. Lu, M. E. Josephson, T. Hyeon, D. H. Kim, H. J. Hwang, *Sci Transl Med* **2016**, *8*, 1.

[13] R. Ma, B. Kang, S. Cho, M. Choi, S. Baik, *ACS Nano* **2015**, *9*, 10876.

[14] S. Choi, J. Park, W. Hyun, J. Kim, J. Kim, Y. B. Lee, C. Song, H. J. Hwang, J. H. Kim, T. Hyeon, D. H. Kim, *ACS Nano* **2015**, *9*, 6626.

[15] R. Libanori, R. M. Erb, A. Reiser, H. Le Ferrand, M. J. Süess, R. Spolenak, A. R. Studart, *Nat Commun* **2012**, *3*, 1.

[16] T. Yang, Y. Zhong, D. Tao, X. Li, X. Zang, S. Lin, X. Jiang, Z. Li, H. Zhu, *2d Mater* **2017**, *4*, DOI 10.1088/2053-1583/aa78cc.

[17] Y. Zhao, B. Wang, J. Tan, H. Yin, R. strain-insensitive bioelectronics featuring brittle materials Huang, J. Zhu, S. Lin, Y. Zhou, D. Jelinek, Z. Sun, K. Youssef, L. Voisin, A. Horrillo, K. Zhang, B. M. Wu, H. A. Coller, D. C. Lu, Q. Pei, S. Emaminejad, *Science* **2022**, *378*, 1222.

[18] J. Li, M. M. Naiini, S. Vaziri, M. C. Lemme, M. Östling, *Adv Funct Mater* **2014**, *24*, 6524.

[19] J. W. T. Seo, J. Zhu, V. K. Sangwan, E. B. Secor, S. G. Wallace, M. C. Hersam, *ACS Appl Mater Interfaces* **2019**, *11*, 5675.

[20] D. McManus, S. Vranic, F. Withers, V. Sanchez-Romaguera, M. Macucci, H. Yang, R. Sorrentino, K. Parvez, S. K. Son, G. Iannaccone, K. Kostarelos, G. Fiori, C. Casiraghi, *Nat Nanotechnol* **2017**, *12*, 343.

[21] F. I. Alzakia, W. Jonhson, J. Ding, S. C. Tan, *ACS Appl Mater Interfaces* **2020**, *12*, 28840.
[22] A. Mazaheri, M. Lee, H. S. J. Van Der Zant, R. Frisenda, A. Castellanos-Gomez, *Nanoscale* **2020**, *12*, 19068.
[23] L. Kong, G. Li, Q. Su, X. Zhang, Z. Liu, G. Liao, B. Sun, T. Shi, *Adv Eng Mater* **2023**, *25*, 1.
[24] T. Y. Kim, J. Ha, K. Cho, J. Pak, J. Seo, J. Park, J. K. Kim, S. Chung, Y. Hong, T. Lee, *ACS Nano* **2017**, *11*, 10273.
[25] F. Ershad, A. Thukral, J. Yue, P. Comeaux, Y. Lu, H. Shim, K. Sim, N. I. Kim, Z. Rao, R. Guevara, L. Contreras, F. Pan, Y. Zhang, Y. S. Guan, P. Yang, X. Wang, P. Wang, X. Wu, C. Yu, *Nat Commun* **2020**, *11*, 1.
[26] Z. Wang, X. Xiao, W. Wu, X. Zhang, Y. Pang, *Biosens Bioelectron* **2024**, *253*, 116150.
[27] Y. Jiang, S. Ji, J. Sun, J. Huang, Y. Li, G. Zou, T. Salim, C. Wang, W. Li, H. Jin, J. Xu, S. Wang, T. Lei, X. Yan, W. Y. X. Peh, S. C. Yen, Z. Liu, M. Yu, H. Zhao, Z. Lu, G. Li, H. Gao, Z. Liu, Z. Bao, X. Chen, *Nature* **2023**, *614*, 456.
[28] R. T. Arwani, S. C. L. Tan, A. Sundarapandi, W. P. Goh, Y. Liu, F. Y. Leong, W. Yang, X. T. Zheng, Y. Yu, C. Jiang, Y. C. Ang, L. Kong, S. L. Teo, P. Chen, X. Su, H. Li, Z. Liu, X. Chen, L. Yang, Y. Liu, *Nat Mater* **2024**, *23*, DOI 10.1038/s41563-024-01918-9.
[29] C. Jeong, G. R. Koirala, Y. H. Jung, Y. S. Ye, J. H. Hyun, T. H. Kim, B. Park, J. Ok, Y. Jung, T. il Kim, *Adv Funct Mater* **2022**, *32*, 1.
